# *Arabidopsis* COG Complex Subunits COG3 and COG8 Modulate Golgi Morphology, Vesicle Trafficking Homeostasis and Are Essential for Pollen Tube Growth

**DOI:** 10.1371/journal.pgen.1006140

**Published:** 2016-07-22

**Authors:** Xiaoyun Tan, Kun Cao, Feng Liu, Yingxin Li, Pengxiang Li, Caiji Gao, Yu Ding, Zhiyi Lan, Zhixuan Shi, Qingchen Rui, Yihong Feng, Yulong Liu, Yanxue Zhao, Chengyun Wu, Qian Zhang, Yan Li, Liwen Jiang, Yiqun Bao

**Affiliations:** 1 College of Life Sciences, Nanjing Agricultural University, Nanjing, People’s Republic of China; 2 School of Life Sciences, Centre for Cell and Developmental Biology and State Key Laboratory of Agrobiotechnology, The Chinese University of Hong Kong, Shatin, New Territories, Hong Kong, China; Brown University, UNITED STATES

## Abstract

Spatially and temporally regulated membrane trafficking events incorporate membrane and cell wall materials into the pollen tube apex and are believed to underlie the rapid pollen tube growth. In plants, the molecular mechanisms and physiological functions of intra-Golgi transport and Golgi integrity maintenance remain largely unclear. The conserved oligomeric Golgi (COG) complex has been implicated in tethering of retrograde intra-Golgi vesicles in yeast and mammalian cells. Using genetic and cytologic approaches, we demonstrate that T-DNA insertions in *Arabidopsis* COG complex subunits, COG3 and COG8, cause an absolute, male-specific transmission defect that can be complemented by expression of COG3 and COG8 from the *LAT52* pollen promoter, respectively. No obvious abnormalities in the microgametogenesis of the two mutants are observed, but *in vitro* and *in vivo* pollen tube growth are defective. COG3 or COG8 proteins fused to green fluorescent protein (GFP) label the Golgi apparatus. In pollen of both mutants, Golgi bodies exhibit altered morphology. Moreover, γ-COP and EMP12 proteins lose their tight association with the Golgi. These defects lead to the incorrect deposition of cell wall components and proteins during pollen tube growth. COG3 and COG8 interact directly with each other, and a structural model of the *Arabidopsis* COG complex is proposed. We believe that the COG complex helps to modulate Golgi morphology and vesicle trafficking homeostasis during pollen tube tip growth.

## Author Summary

In the pistils of flowering plants, pollen tubes elongate at the tips to deliver the male gametes to the egg cells for fertilization. The tip growth of pollen tube is due to the deposition of cell membranes and wall materials at a restricted tip area of the plasma membrane. Vesicle trafficking events occurred at the extreme tips have been shown to be required for the coordinated tip growth, while other trafficking pathways are less well characterized. On the other hand, little is known about the molecular mechanisms and the physiological impact of Golgi trafficking and Golgi structure maintenance in plant cells. COG complex is a vesicle tethering factor supposed to mediate intra-Golgi retrograde transport in mammalian and yeast cells. By characterization of two plant COG complex subunits COG3 and COG8, we revealed their roles in Golgi transport and Golgi structure maintenance, which are essential for pollen tube polar growth. These results deepened our understandings on pollen tube growth regulation, and the molecular mechanisms of Golgi trafficking and Golgi morphology maintenance in plant cells.

## Introduction

In flowering plants, pollen tubes grow through the style and deliver male gametes to ovules through highly polarized growth of the tips caused by cell expansion occurring exclusively at the apex [1]. Pollen tubes can grow rapidly under both *in vivo* and *in vitro* conditions where actomyosin-dependent reverse fountain-like cytoplasmic streaming efficiently drives vesicles into the clear zone of a growing pollen tube [2]. Most of these vesicles fuse with the apex membrane and deposit cell wall materials, membrane lipids, and proteins to support growth. This deposition must be regulated temporally and spatially to balance turgor pressure and cell wall extensibility [3, 4]. The massive amount of exocytosis that occurs at the pollen tube tip was calculated to exceed the requirements for maintaining growth rates, and was suggestive of underlying endocytosis and recycling processes [5, 6, 7, 8]. How these processes are coordinated during pollen tube growth remains unclear.

Key components of the vesicle trafficking machinery which operates during pollen tube growth are being characterized [4, 9]. Small GTPase NtRAB11B labeled transport vesicles in the apical inverted cone of the growing pollen tube, and play a role in secretory vesicle delivery and possibly vesicle recycling [10]. A knockout mutation of the *Arabidopsis* pollen-specific *RABA4D* gene impaired selective targeting of cell wall materials and pollen tube guidance [11]. In addition to Rab GTPases, several mutants of *Arabidopsis* exocyst subunits exhibited short and swollen pollen tubes, and the tip-localization patterns of EXO70A1, SEC6, and SEC8 were suggestive of a role in polarized exocytosis or recycling at the tips [12, 13, 14]. Recently, pollen-specific GNL2 was shown to be essential for pollen tube tip growth based on its necessary role in polar recycling [15]. These results demonstrated that the tip-focused delivery of exocytic and recycling vesicles is crucial for polarized and directional pollen tube growth. In addition, a dominant negative form of tobacco NtRAB2 blocked secretory protein trafficking and arrested pollen tube growth [16] indicative of the importance of the endoplasmic reticulum (ER)-Golgi early secretory pathway.

The ER-to-Golgi secretion pathway mediated by the coat protein complex II (COPII) is thought to be counter-balanced by COPI-mediated retrograde trafficking [17, 18]. In yeast and mammalian cells, the conserved oligomeric Golgi (COG) complex, which is an octameric tethering complex, is involved in COPI-mediated, intra-Golgi retrograde transport of Golgi-resident proteins such as glycosyltransferases, which are enzymes that glycosylate proteins and lipids [19, 20, 21, 22]. In addition, the COG complex is required for the integrity of the mammalian Golgi apparatus [21, 23].

Homologs of each of the COG complex subunits have been identified in the *Arabidopsis* genome [24] and its presumed partners in COPI-mediated intra-Golgi trafficking, such as COPI vesicles, were observed predominantly at the periphery of *cis-* and *medial-*cisternae of plant Golgi [25, 26]. Moreover, COPI components and the GTPase ADP ribosylation factor 1 (Arf1), which is responsible for COPI coat assembly and dissociation, have been identified in plants [17, 27, 28, 29, 30]. COPI is essential for COPII-mediated anterograde trafficking in tobacco leaf epidermal cells [17]. Aside from the involvement of a presumed association between COPI and the COG complex in intra-Golgi trafficking, little is known regarding the effects of COG complex disruption on COPI-mediated trafficking in plants.

The Golgi apparatus in plant cells consists of numerous individual stacks moving over the ER with the aid of actin-myosin motors. This arrangement differs from the perinuclear Golgi stacks which are arranged side-by-side in mammalian cells [18, 31, 32]. Understanding the mechanisms by which the plant Golgi structure is maintained, despite its rapid movement and exchange of proteins with other organelles, is important. Long coiled-coil protein golgins have been implicated in the maintenance of Golgi integrity in mammalian cells by linking adjacent membranes in vesicle docking and by linking adjacent cisternae [33]. Among plant golgin homologs that localize to the Golgi [24, 34, 35, 36], Atp115/GC6 [36] and AtCASP [34, 35] may function as vesicle tethering factors, but the roles of golgins in plant Golgi structure maintenance have not been demonstrated, nor have the roles of the multi-subunit tethering complexes, TRAPPI/II and COG complex [18].

The *Arabidopsis embryo yellow* (*eye*) mutant, in which the *COG7* gene is disrupted, exhibits abnormal embryo color and development, mislocalization of ERD2 to the ER, and alterations in the size of the Golgi apparatus [37]. Moreover, the COG complex was implicated in penetration resistance of barley to barley powdery mildew fungus [38]. Our current understanding of COG complex function in plant development remains limited. How Golgi functions dependent on COG complex coupled to plant development, particularly pollen tube tip growth is a very interesting question to answer.

## Results

### Mutations in *COG3* and *COG8* genes cause male sterility

**Fig 1:**
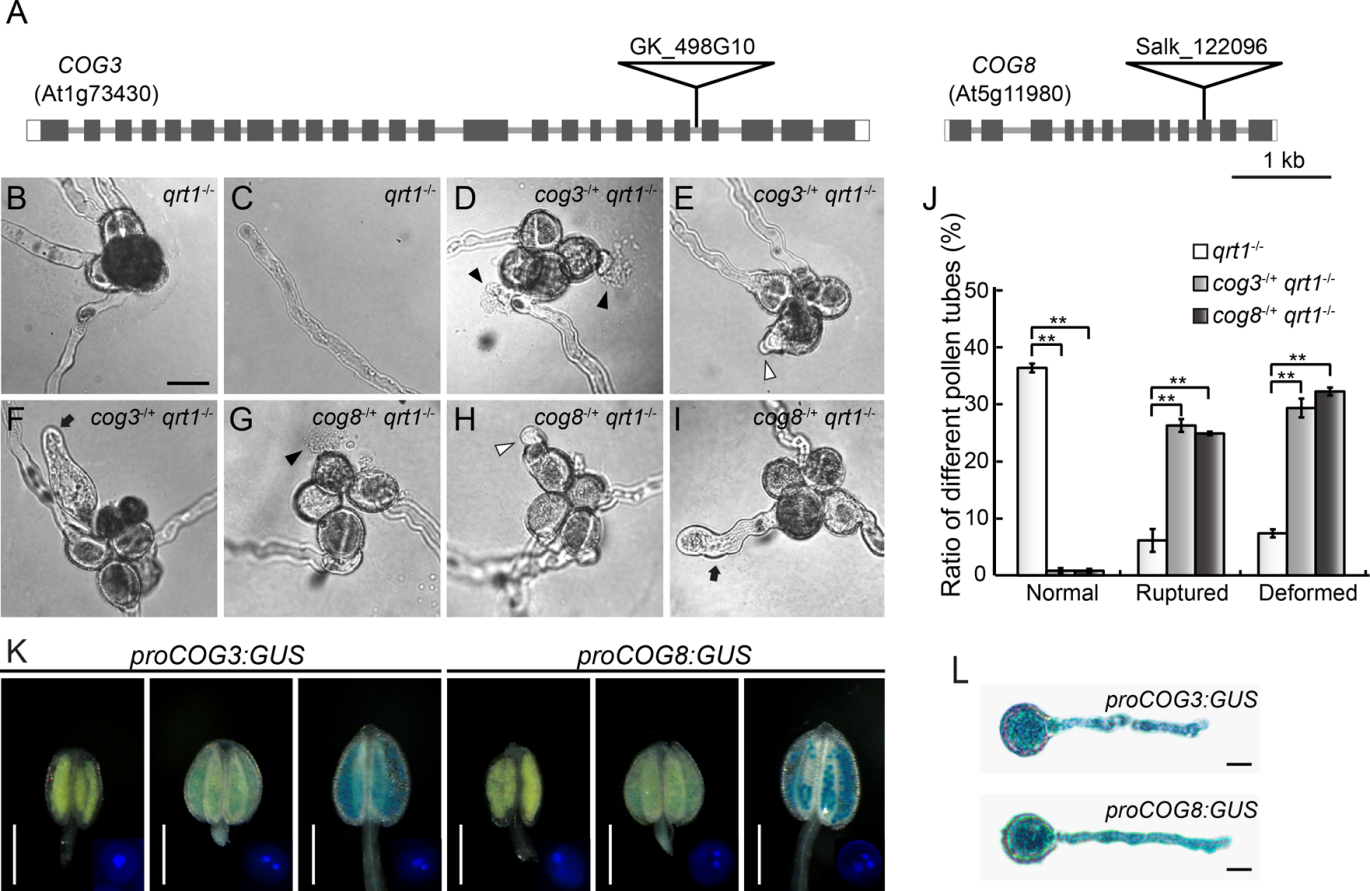
Mutations in the *COG3* and *COG8* genes cause *in vitro* pollen tube growth defects. (**A**) Exon/intron structures and the positions of T-DNA insertions in the *COG3* and *COG8* genes. (**B**) A *qrt1*^*-*/*-*^ quartet with four growing pollen tubes. (**C**) A normal pollen tube from a *qrt1*^*-*/*-*^ quartet. (**D**) to (**F**) Pollen tubes of *cog3*^*-*/*+*^ *qrt1*^*-*/*-*^ quartets. (**G**) to (**I**) Pollen tubes of *cog8*^*-*/*+*^ *qrt1*^*-*/*-*^ quartets. (**D**) to (**I**) Ruptured (black arrowheads), short (white arrowheads), and swollen and wavy (black arrow) pollen tubes were observed in both mutants after 6 h of *in vitro* germination. (**J**) Quantitative comparison of pollen tube defects of *qrt1*^*-/-*^ (n = 698), *cog3*^*-/+*^ *qrt1*^*-/-*^ (n = 712), and *cog8*^*-/+*^ *qrt1*^*-/-*^ (n = 716). Short, swollen, and wavy pollen tubes were classified as deformed. Values represent the means ± SD, ** indicates *P*< 0.001 by Student’s *t* test. (**K**) *COG3* and *COG8* start to express at the bicellular stage of pollen development. DAPI staining of pollen in GUS-stained anthers at the uni-, bi-, or tri-cellular stage is shown in the right corner of each image. (**L**) Both *COG3* and *COG8* are expressed in pollen tubes. Bars = 20 μm in (**B**), 0.5 mm in (**K**), and 10 μm in (**L**).

*COG3* is interrupted in intron 21 by a T-DNA insertion in the *Arabidopsis* line GK_498G10, while *COG8* is interrupted by a T-DNA insertion in exon 10 in the Salk_122096 line (Fig 1A). No homozygous *cog3* or *cog8* mutant plants were identified using PCR-based genotyping with combinations of different gene-specific primers and a T-DNA border primer (n>300). The progeny from self-pollinated *cog3*^*-/+*^ and *cog8*^*-/+*^ plants all segregated at a ratio of roughly 1:1 instead of the expected 3:1 ratio. For 134 heterozygous and 165 wild-type progeny of the *cog3*^*-/+*^ mutant, a χ^2^ test for a 1:1 ratio gave a value of 3.21, *P* < 0.05. For 181 heterozygous and 174 wild-type progeny of the *cog8*^*-/+*^ mutant, a χ^2^ test for a 1:1 ratio gave a value of 0.14, *P* < 0.05 (Table 1). Pollination of wild-type plants with pollen from *cog3*^*-/+*^ or *cog8*^*-/+*^ mutant plants resulted only in offspring with no insertion (Table 1) indicating that the two mutations cannot be transmitted by the male gametophytes. In contrast, when *cog3*^*-/+*^ and *cog8*^*-/+*^ were used as recipients in crosses with wild-type pollen, approximately 45% [113/(113+138)] of *cog3*^*-/+*^ and 44% [94/(94+120)] of *cog8*^*-/+*^ progeny contained the T-DNA insertion indicating that the T-DNA insertions in *COG3* and *COG8* had no discernible effect on female gametophyte transmission.

**Table 1:** Genetic analysis of *cog3*^*-/+*^ and *cog8*^*-/+*^ mutants.

Cross (Female × male)	PCR^+^^a^	PCR^-^^b^	Ratio	TE_F_^c^	TE_M_
*cog3*^*-/+*^ × *cog3*^*-/+*^	134	165	0.81	NA^d^	NA
*cog3*^*-/+*^ × *COG3*^*+/+*^	113	138	0.82	82%	NA
*COG3*^*+/+*^ × *cog3*^*-/+*^	0	249	0	NA	0
*cog8*^*-/+*^ × *cog8*^*-/+*^	181	174	1.04	NA	NA
*cog8*^*-/+*^ × *COG8*^*+/+*^	94	120	0.79	79%	NA
*COG8*^*+/+*^ × *cog8*^*-/+*^	0	220	0	NA	0

^a^progeny positive for the PCR analysis in the *cog3*^*-/+*^ or *cog8*^*-/+*^ mutant background.

^b^progeny negative for the PCR analysis in the *cog3*^*-/+*^ or *cog8*^*-/+*^ mutant background.

^c^TE, transmission efficiency; TE = (progeny with PCR^+^/progeny with PCR^-^) ×100%; TE_F_ and TE_M_, female and male transmission efficiency, respectively.

^d^NA, not applicable.

### *cog3* and *cog8* mutants are defective in pollen tube growth *in vitro* and *in vivo*

The failure of male transmission in the *cog3*^*-/+*^ and *cog8*^*-/+*^ mutants was indicative of defective male gametophyte development. We introduced the *cog3* and *cog8* alleles into a *quartet* (*qrt1*) mutant background which allowed us to perform comparative analysis of four meiotic products because they do not separate from each other [39]. A quartet from a resulting *cog3*^*-/+*^ *qrt1*^*-/-*^ plant had two *cog3 qrt1* (mutant) and two *qrt1* (wild-type) pollen grains. The same genotypic frequency applied to a *cog8*^*-/+*^ *qrt1*^*-/-*^ quartet. As shown in S1 Fig, Alexander staining showed that almost all *cog3*^*-/+*^ *qrt1*^*-/-*^ and *cog8*^*-/+*^ *qrt1*^*-/-*^ quartets had four viable pollen grains (S1 Fig). Scanning electron microscopy (SEM) and 4′,6-diamidino-2-phenylindole (DAPI) staining revealed that these pollen grains were normal in appearance (S1 Fig) and contained two sperm cells and one vegetative cell (S1 Fig). Therefore, pollen grain development was not affected by the *cog3* or *cog8* mutations.

We examined *in vitro* germination and pollen tube growth of *cog3*^*-*/+^ *qrt1*^*-*/-^ and *cog8*^*-*/+^ *qrt1*^*-*/-^ quartets after incubation on solid media for 6 h at 22°C. No more than two normal pollen tubes were observed in the mutants (Fig 1D–1I) in contrast with the wild-type (Fig 1B), while many ruptured, short, swollen, and wavy pollen tubes were observed in the mutants (Fig 1D–1I), but not in the wild-type (Fig 1C). To analyze the defective pollen tube growth statistically, we established counting criteria. Quartets producing zero or one pollen tube were excluded. For quartets with two normal pollen tubes, the phenotypes of the other two pollen were counted. For example, in Fig 1D, the two pollen with normal tubes were not counted and the two pollen with burst tubes were counted as ‘ruptured’. Approximately 26% of *cog3*^*-/+*^ and 25% of *cog8*^*-/+*^ counted pollen tubes ruptured immediately after germination or after growth. In addition, approximately 29% of *cog3*^*-/+*^ and 32% of *cog8*^*-/+*^ counted pollen tubes were short or swollen and wavy and were classified as ‘deformed’. Therefore, the amounts of both ‘ruptured’- and ‘deformed’- types of pollen increased significantly (*P* < 0.001) (Fig 1J). In the meantime, the germination rates of counted pollen from *cog3*^*-/+*^ *qrt1*^*-/-*^ (n = 712) and *cog8*^*-/+*^ *qrt1*^*-/-*^ (n = 716) were approximately 56 and 58%, respectively, which was comparable to that of *qrt1*^*-/-*^ pollen (50%). The same types of defects with similar ratios (S2 Fig) were observed for *cog3*^*-*/+^ and *cog8*^*-*/+^ heterozygotes after germination for 4 h on solid media and the mutant pollen tubes grew significantly slower than the wild-type (S3 Fig). Taken together, *in vitro* germination assays indicated that mutations in *COG3* and *COG8* genes caused unstable, slow growing, and morphologically abnormal pollen tubes.

The *COG3* and *COG8* genes exhibited similar patterns of ubiquitous expression as determined by real time (RT)-PCR (S4 Fig), and β-glucuronidase (GUS) assays performed in 13 and 5 independent transgenic plant lines harboring the *proCOG3*:*GUS* and *proCOG8*:*GUS* constructs, respectively (*proCOG3*: 1057 bp; *proCOG8*: 504 bp in relation to ATG, S4 Fig). Both genes were not expressed during pollen development until the binucleated stage and their expression levels increased markedly in trinucleated pollen (Fig 1K) and pollen tubes (Fig 1L), which was consistent with the observed phenotypes.

**Fig 2:**
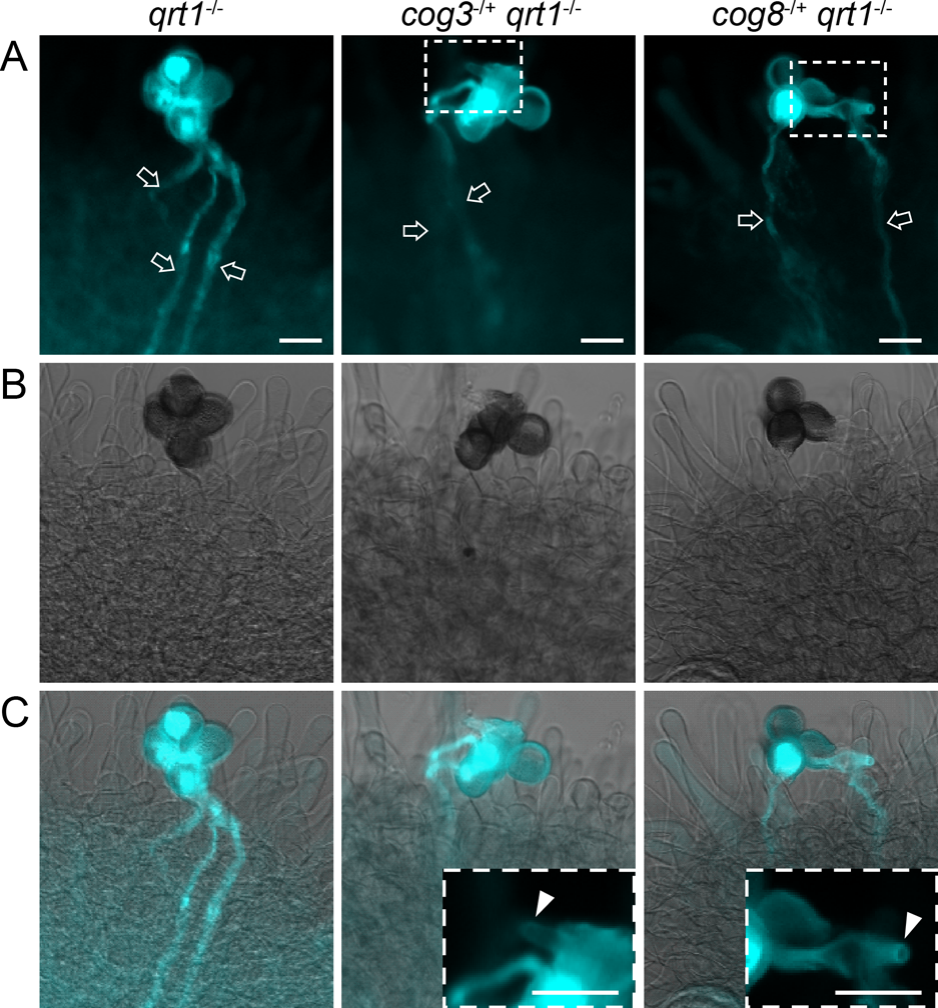
*In vivo* pollen tube growth is defective in *cog3* and *cog8* mutants. (**A**) to (**C**) Aniline blue staining of quartets germinated *in vivo*. (**A**) In contrast with *qrt1*^*-*/-^ quartets, no more than two normal pollen tubes (arrow) were observed in the mutants. (**B**) Bright field images of (**A**). (**C**) Merged images of (**A**) and (**B**). Insets are magnified images of the selected areas in (**A**) showing that the mutant pollen tubes were arrested at the surfaces of stigma cells (arrowheads). Bars = 20 μm.

We examined *in vivo* pollen tube growth using artificial pollination of *cog3*^*-*/+^ *qrt1*^*-*/-^ and *cog8*^*-*/+^ *qrt1*^*-*/-^ pollen grains onto *ms1*^*-*/-^ mutant pistils. The *ms1* mutation affects the early stage of pollen development and thus provides readily available unpollinated stigmas [40]. After 16 h of pollination, approximately 68% (n = 112) of *qrt1*^*-*/*-*^ quartets grew three or four normal pollen tubes into the pistil as revealed by aniline blue staining (Fig 2A). In contrast, none of the *cog3*^*-*/+^ *qrt1*^*-*/-^ (n = 73) or *cog8*^*-*/+^ *qrt1*^*-*/-^ (n = 56) quartets germinated three or four normal pollen tubes; the majority germinated two normal pollen tubes plus one or two aberrant pollen tubes which did not grow into pistils (Fig 2B and 2C). *COG3* and *COG8* mutations disrupted pollen tube growth both *in vitro* and *in vivo*.

### *cog3* and *cog8* mutants were rescued by *COG3* and *COG8*, respectively

We transformed a construct containing a pollen-specific *LAT52* promoter driving expression of *COG3* or *COG8* coding sequences fused to green fluorescent protein (GFP) into *cog3*^*-/+*^ and *cog8*^*-/+*^ plants and obtained three *cog3*^*-*/+^ *proLAT52*:*COG3-GFP*^*-/+*^ and two *cog8*^*-/+*^ *proLAT52*:*COG8-GFP*^*-*/+^ transgenic lines (Table 2). PCR-aided genotyping using genomic DNA extracted from progeny of these lines demonstrated that the ratio of plants with T-DNA allele increased from approximately 1:1 to 1.5:1 (Table 2). One explanation for the increased ratios could be that the pollen defects of *cog3* and *cog8* were completely complemented by *proLAT52*:*COG3*-*GFP* and *proLAT52*:*COG8*-*GFP*, respectively, but the rescued plants were embryo- and/or seedling-lethal (see below). Indeed, the pollen tube growth of *cog3*^*-*/+^ *proLAT52*:*COG3-GFP/proLAT52*:*COG3-GFP* and *cog8*^*-/+*^ *proLAT52*:*COG8-GFP/proLAT52*:*COG8-GFP* plants returned to normal (S2 Fig). In addition, we obtained four *cog8*^*-*/+^ lines complemented with *COG8* genomic DNA (*cog8*^*-/+*^ *gCOG8*^*-/+*^) in which the male transmission efficiency increased to approximately 2:1 indicating a complete complementation (S1 Table). Indeed, among the progeny, *cog8*^*-/-*^ *gCOG8/gCOG8* pollen tubes and plants grew normally as the wild-type (S5 Fig). These results showed that loss function of *COG3* and *COG8* was responsible for the mutant phenotypes of *cog3*^*-/+*^ and *cog8*^*-/+*^ plants, respectively.

**Table 2:** Complementation analysis of *cog3*^*-/+*^ and *cog8*^*-/+*^ mutants.

Self-cross	PCR^+^^a^	PCR^-^^b^	Ratio^c^	*X*^*2*^ (For 1.5:1)
*cog3*^*-/+*^	134	165	0.81	NA
*cog3*^*-/+*^ *ProLAT52*:*COG3-GFP*^*-/+*^ (line1)	137	87	1.57	0.125
*cog3*^*-/+*^ *Pro LAT52*:*COG3-GFP*^*-/+*^ (line2)	117	73	1.60	0.197
*cog3*^*-/+*^ *Pro LAT52*:*COG3-GFP*^*-/+*^ (line3)	127	77	1.65	0.432
*cog8*^*-/+*^	181	174	1.04	NA
*cog8*^*-/+*^ *ProLAT52*:*COG8-GFP*^*-/+*^ (line1) (line…(line(line.)	125	79	1.58	0.138
*cog8*^*-/+*^ *ProLAT52*:*COG8-GFP*^*-/+*^ (line2)	130	70	1.86	2.081

^a^progeny positive for the PCR analysis in the *cog3*^*-/+*^ or *cog8*^*-/+*^ mutant background.

^b^progeny negative for the PCR analysis in the *cog3*^*-/+*^ or *cog8*^*-/+*^ mutant background.

Ratio = PCR^+^/PCR^-^.

^c^*X*^*2*^ test indicated that the segregation ratio is coincident with expected 1.5:1 (P<0.05).

^d^g is the abbreviation for genomic DNA.

1.86

### COG3 and COG8 are required for sporophytic development

**Fig 3:**
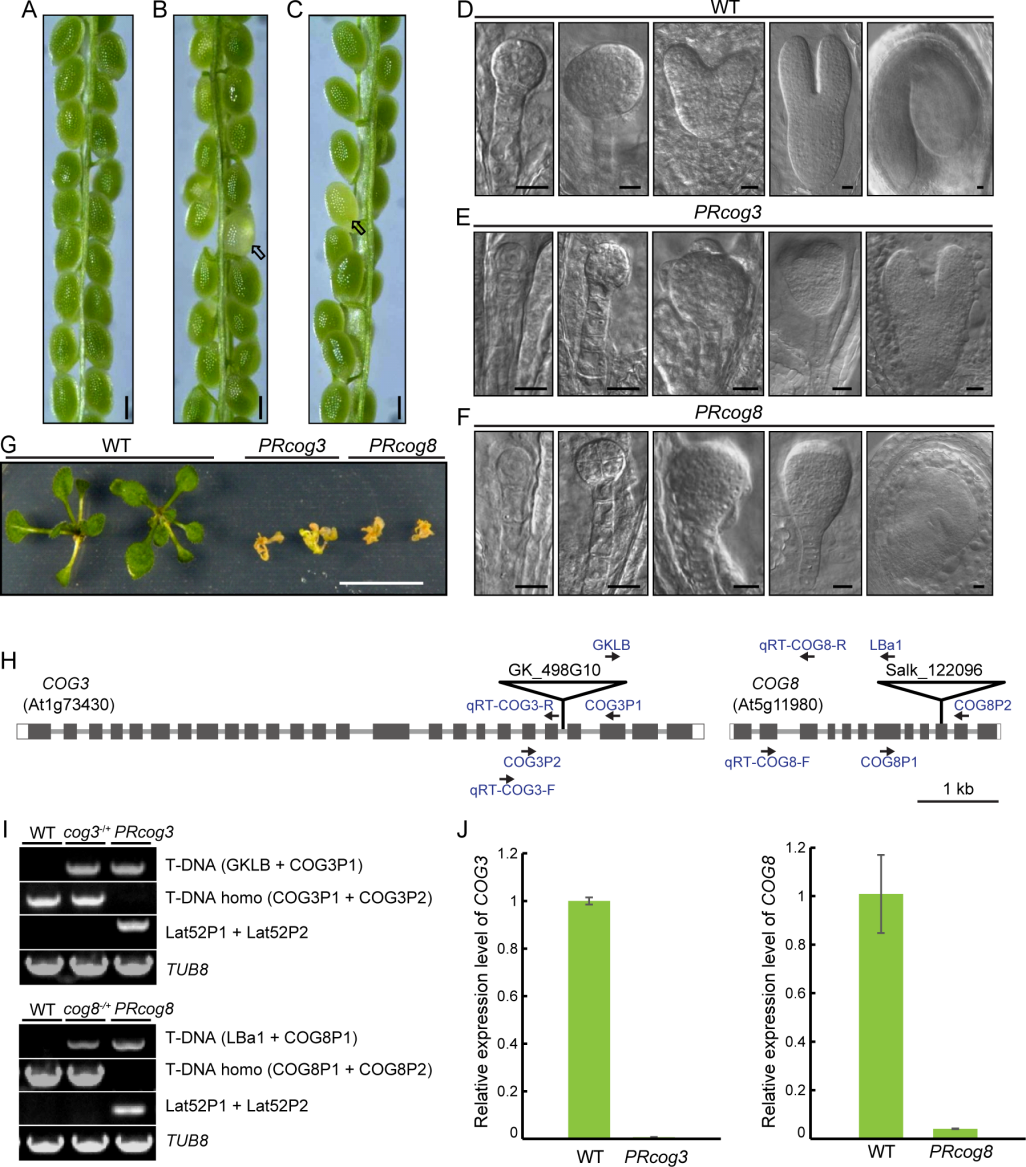
COG3 and COG8 are required for sporophytic development. (**A**) A silique from wild-type plants. (**B**) A silique from *cog3*^*-*/+^ *proLAT52*:*COG3-GFP*/*proLAT52*:*COG3-GFP* plants. (**C**) A silique from *cog8*^*-*/+^ *proLAT52*:*COG8-GFP*/*proLAT52*:*COG8-GFP* plants. (**D**) Wild-type embryos at various developmental stages. (**E**) Abnormal embryos from *PRcog3* seeds. (**F**) Abnormal embryos from *PRcog8* seeds. (**G**) *PRcog3* and *PRcog8* seedlings died approximately 18 days after germination. (**H**) Location of primers used for genotyping and quantitative RT-PCR. Note that primers *Lat52P1* and *Lat52P2* located on the vector are not shown here. (**I**) Genotyping of *PRcog3* and *PRcog8* mutants. (**J**) Quantitative RT-PCR analysis showed that *COG3* and *COG8* expressions were barely detectable in *PRcog3* and *PRcog8* mutants, respectively. *TUB8* was used as an internal control. Genomic DNA and total RNA were extracted from 10^th^-day seedlings. Arrows in (**B**) and (**C**) indicate white seeds. Bars = 250 μm in (**A**) to (**C**), 10 μm in (**D**) to (**F**), 1 cm in (**G**).

Because *COG3* and *COG8* gene expression was not limited to pollen (S4 Fig), additional phenotypes in *cog3*^*-*/+^ *proLAT52*:*COG3-GFP/proLAT52*:*COG3-GFP* and *cog8*^*-/+*^ *proLAT52*:*COG8-GFP/proLAT52*:*COG8-GFP* plants were explored. White seeds (approximately 15%) were noticed in the siliques of two randomly selected independent lines for each genotype, but were not present in wild-type siliques (Fig 3A–3C and S2 Table). Embryo development in the white seeds was delayed from the early stages, was arrested at heart stages, or resulted in abnormal curled cotyledons (Fig 3D–3F). These defective seeds exhibited shrinkage at approximately13^th^-day after fertilization. In addition, approximately 10% of seedlings died at about 18^th^-day after germination for each genotype (Fig 3G and S2 Table). Therefore, the portion of both mutants which were embryo- and seedling-lethal accounted for approximately 25% of the corresponding populations. Based on the genotyping (Fig 3H and 3I) and quantitative RT-PCR results (Fig 3H and 3J), the 25% of each population are probably sporophytic *cog3* or *cog8* homozygotes, and designated as pollen rescued *cog3* (*PRcog3*: *cog3*^*-*/-^ *proLAT52*:*COG3-GFP/proLAT52*:*COG3-GFP*), and pollen rescued *cog8* (*PRcog8*: *cog8*^*-*/-^ *proLAT52*:*COG3-GFP/proLAT52*:*COG3-GFP*) respectively. In short, the COG complex plays a role in sporophytic development as well.

### COG3 and COG8 are localized to the Golgi apparatus in pollen

**Fig 4:**
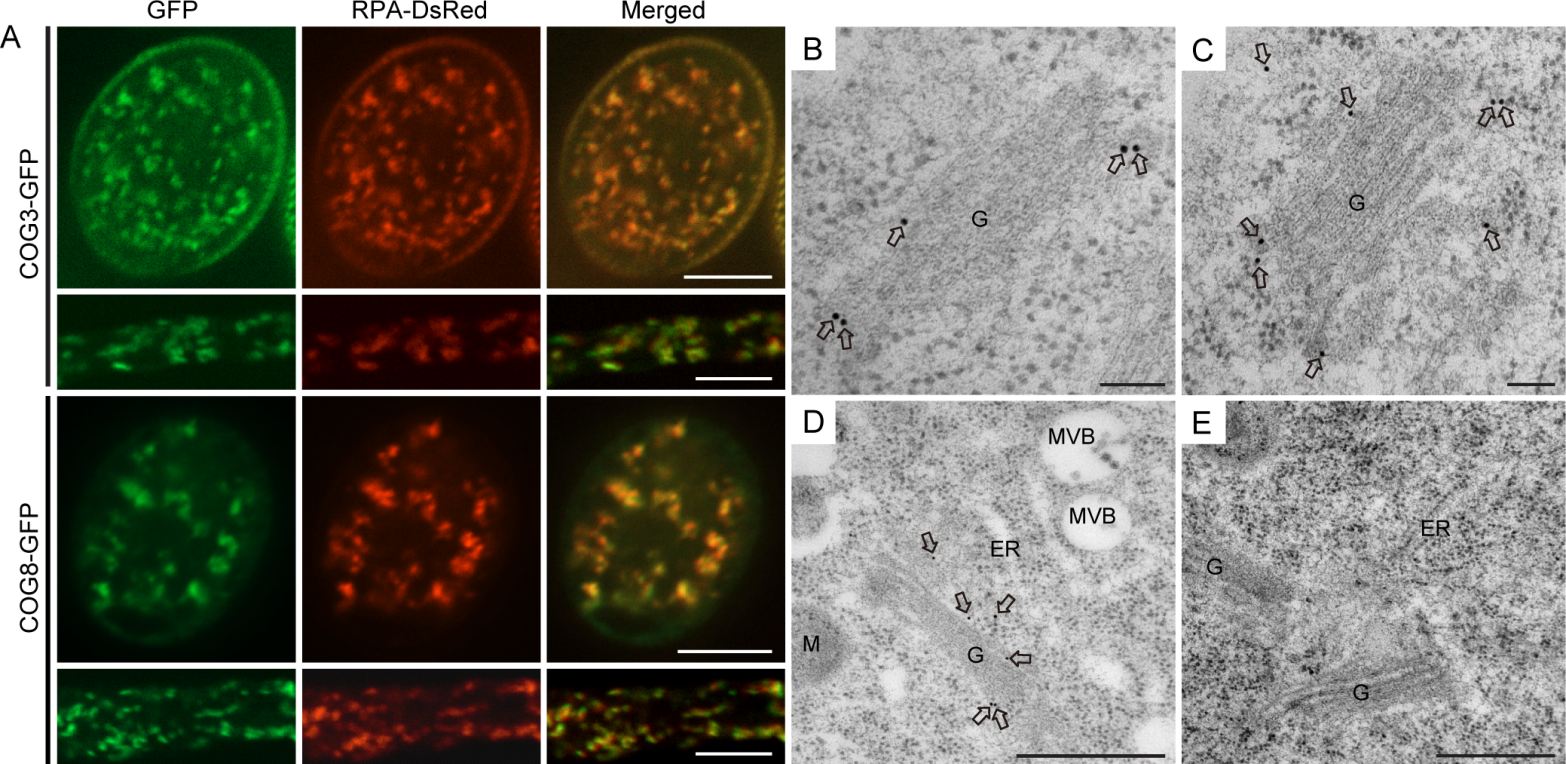
COG3 and COG8 localize to the Golgi apparatus. (**A**) Confocal microscopy images showing that COG3-GFP and COG8-GFP signals largely overlap with the Golgi marker RPA-DsRed in pollen grains and tubes. (**B**), (**C**) and (**D**) Anti-GFP antibody labeling on the ultrathin sections prepared using high-pressure frozen/freeze-substituted root cells of transgenic plants expressing *pro35S*:*COG8-GFP*. (**B**) and (**C**) COG8 proteins were found mainly at the periphery of the Golgi apparatus (arrows). (**D**) COG8 proteins localized to the Golgi body but not to other organelles shown in the image. G: Golgi apparatus; M: Mitochondria; ER: Endoplasmic Reticulum; MVB: Multivesicular body. (**E**) The negative control using the secondary antibodies only. Bars = 10 μm in (**A**), 100 nm in (**B**) and (**C**), 500 nm in (**D**) and (**E**).

Determining the localization of COG3 and COG8 proteins in pollen would increase our understanding of the relationship between COG complex function and pollen tube tip growth. The *proLAT52*:*COG3*-GFP and *proLAT52*:*COG8*-GFP fusion proteins were functional because they rescued their corresponding mutants (S2 Fig and Table 2); thus, their subcellular localization in pollen should reflect COG3 and COG8 localization. COG3-GFP and COG8-GFP exhibited punctate fluorescence signals in pollen grains and tubes (Fig 4A) and moved with the reverse fountain-type of cytoplasmic streaming as shown by live cell imaging (S1 and S2 Movies), suggestive that they are associated with the Golgi apparatus. To determine the nature of the compartments with punctuate signals, the COG3-GFP and COG8-GFP plants were crossed with transgenic lines expressing the *LAT52* promoter-driven Golgi marker protein RPA with its C-terminus fused to DsRed (RPA-DsRed) [41, 42] or ER marker mCherry-HDEL [43]. The green fluorescence signals of COG3-GFP and COG8-GFP exhibited the same localization pattern as the red fluorescence signals of RPA-DsRed (Fig 4A) but not of mCherry-HEDL (S6 Fig) indicating that COG3 and COG8 were indeed present in the Golgi apparatus before and after germination.

To verify the subcellular localization of COG8, we generated *pro35S*:*COG8-GFP Arabidopsis* transgenic plants. Immunogold labeling with anti-GFP antibodies revealed that COG8-GFP was located at the Golgi apparatus, in particular, it was targeted to the periphery of the Golgi cisternae (Fig 4B and 4C), which resembled γ-COP localization [25]. While it was not present at other organelles, such as the ER and the multi-vesicular body (MVB) (Fig 4D). And applying only the secondary antibodies gave no signal (Fig 4E).

### Altered morphology of Golgi labeled by GFP-EMP12 and GAUT14-GFP in *cog3* and *cog8* pollen

**Fig 5:**
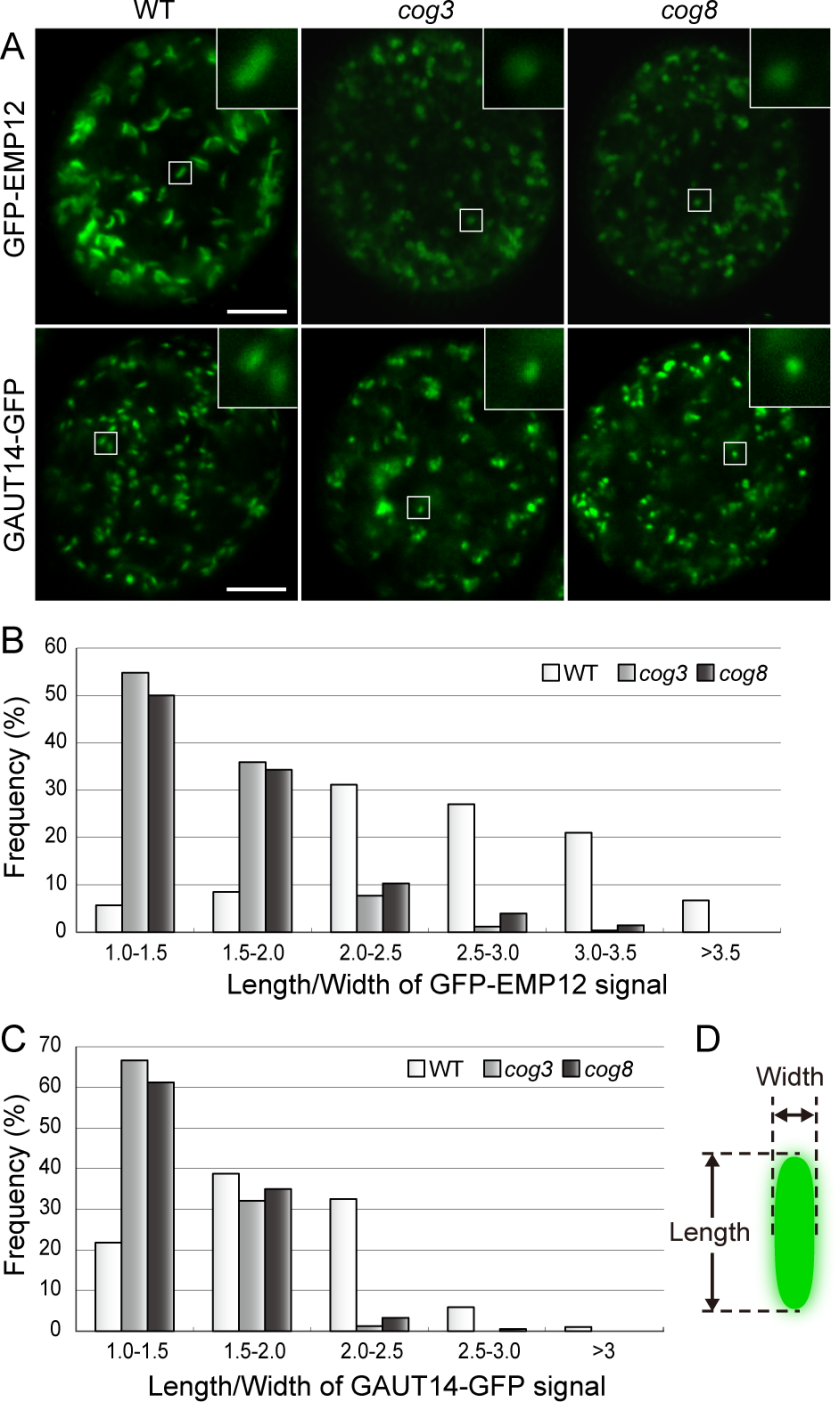
Altered Golgi morphology in *cog3* and *cog8* mutant pollen grains revealed by GFP-EMP12 and GAUT14-GFP fluorescence. (**A**) Altered Golgi apparatus morphology in *cog3* and *cog8* pollen grains. GFP-EMP12 and GAUT14-GFP served as Golgi markers. Insets are 4× magnified images of the selected areas. (**B**) and (**C**) The distribution of Golgi body length to width ratios determined from GFP-EMP12 and GAUT14-GFP signals. For each genotype, all distinguishable Golgi bodies in five pollen grains were measured. (For GFP-EMP12: wild-type, n = 224; *cog3*, n = 259; *cog8*, n = 203. For GAUT14-GFP: wild-type, n = 289; *cog3*, n = 162; *cog8*, n = 183). **(D)** Diagram of a Golgi body with length and width indicated. Bars = 5 μm in (**A**).

Because COG3 and COG8 localized to the Golgi in pollen (Fig 4), we analyzed the morphology of the Golgi apparatus in *cog3*^*-/+*^ and *cog8*^*-/+*^ plants using confocal microscopy. The Golgi marker constructs *proUBQ10*:*GFP-EMP12* [44] and *proPPME1*:*GAUT14-GFP* [45] were first transformed into wild-type plants, respectively. Individual GFP-EMP12 fluorescence spots looked sharp, discrete and well-distributed in wild-type pollen grains, appeared either as discs or bars depending on the viewing angle (Fig 5A), typical of Golgi morphology [46]. However, after crossing, approximately half of pollen grains (*cog3*^*-*/+^: 67/114; *cog8*^*-*/+^: 83/148) showing fuzzy, rounded Golgi bodies tend to clump together in a blurry background, which were readily discernible from wild-type, and were counted as mutant pollen grains (Fig 5A). Length to width ratio (length/width) of GFP-EMP12 signal of all distinguishable Golgi bodies in five pollen grains was measured for each genotype (Fig 5B and 5D). Pollen with that in a range of 1.0 to 2.0 is about 14% in wild-type, 91% in *cog3*, 84% in *cog8*. In contrast, pollen with that of 2.0 to >3.5 is about 86% in wild-type, 9% in *cog3*, 16% in *cog8* (Fig 5B). These data demonstrated that Golgi morphology was dramatically altered in *cog3* and *cog8* pollen. GAUT14-GFP-labeled Golgi [45] also exhibited similar morphological disruption in about half of the pollen grains (*cog3*^*-*/+^: 63/143; *cog3*^*-*/+^: 65/138), although GAUT14-GFP fluorescent bars were generally shorter than those of GFP-EMP12 in wild-type cells (Fig 5A and 5C).

### Change of Golgi apparatus morphology in *cog3* and *cog8* pollen

**Fig 6:**
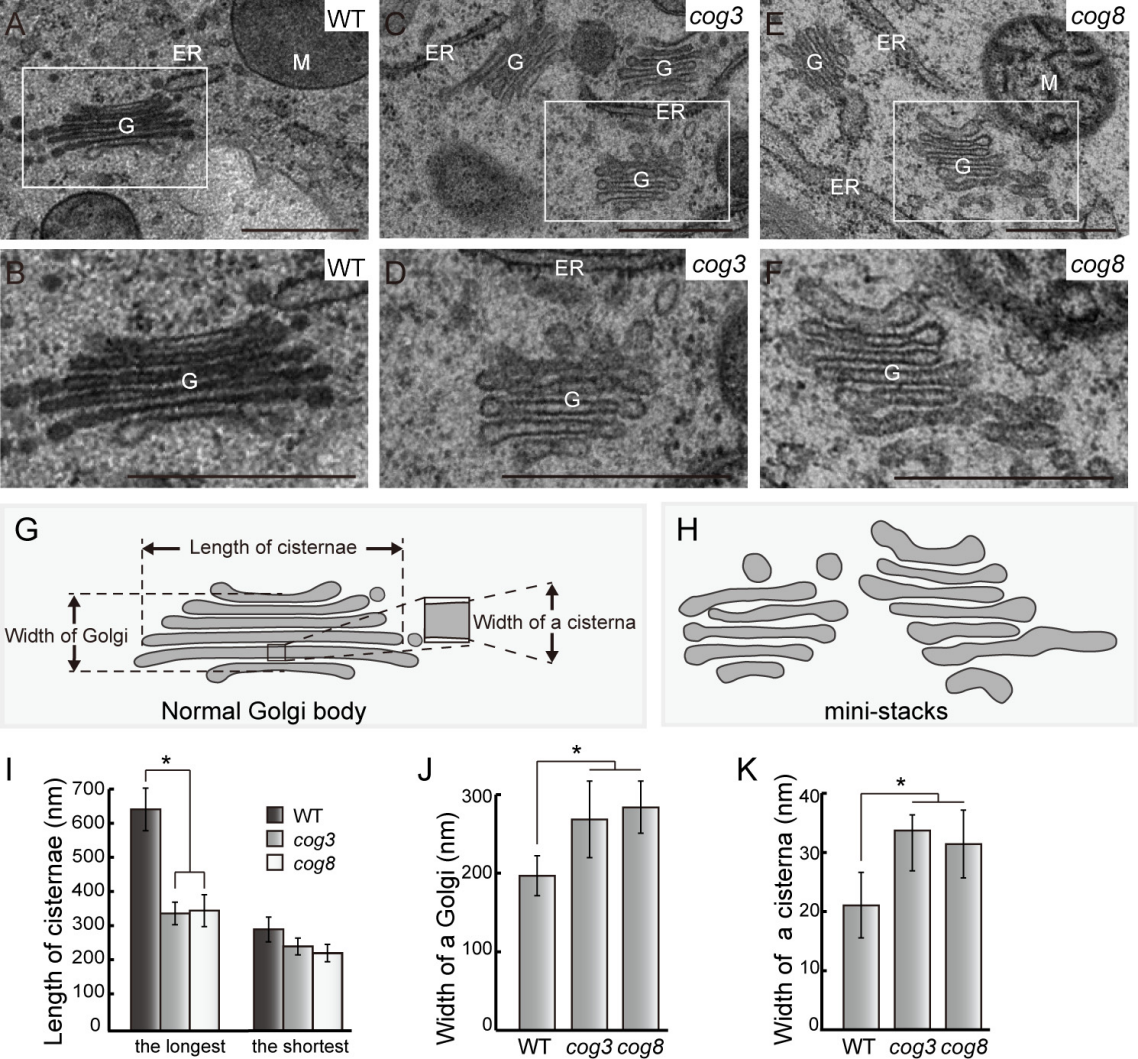
Disruption of the Golgi apparatus structure in *cog3* and *cog8* mutant pollen grains based on TEM observation. (**A**) and **(B)** Structure of the Golgi apparatus in wild-type (*qrt1*) pollen grains. (**C**) to (**F**) Golgi mini-stacks in *cog3* and *cog8* mutant pollen grains. (**G**) Schematic presentation of parameters used for statistical analysis of Golgi mini-stacks. (**H**) Diagrams of Golgi mini-stacks in *cog3* and *cog8* pollen grains respectively. (**I**) to (**K**) Quantification of the abnormalities of Golgi mini-stacks. n> 35 Golgi bodies were measured for each genotype. Values represent the means ± SD. * means *P*< 0.05 by Student’s *t* test. Bar = 500 nm for (**A**) to (**F**).

To further explore abnormalities in Golgi morphology, ultrathin sections of high-pressure frozen/freeze-substituted *cog3*^*-*/+^ *qrt1*^*-*/-^ and *cog8*^*-*/+^ *qrt1*^*-*/-^ pollen grains were used for transmission electron microscopy (TEM) observation. Wild-type and mutant pollen within a quartet were readily discernible based on differences in Golgi morphology. In wild-type pollen, the Golgi apparatus exhibited normal morphology with clear *cis*-*trans* polarity (Fig 6A and 6B), while in *cog3* and *cog8* pollen, all Golgi observed were disrupted, and were named mini-stacks as shown in Fig 6C–6F and as is more clearly illustrated in Fig 5H. In this case, the number of cisternae (five or six) was not significantly different from that of wild-type, but a few obvious changes were noticed and characterized. First, clear recognition of the *cis*-*trans* polarity was difficult. Second, the lengths of the mini-stacks cisternae were reduced significantly with the longest cisterna lengths decreased from approximately 638 nm to 339 nm in *cog3* and to 347 nm in *cog8* and the shortest cisterna lengths decreased from approximately 293 nm to 244 nm in *cog3* and to 255 nm in *cog8* (Fig 6I). Concomitantly, the widths of the mini-stack cisternae increased from approximately 195 nm to 267 nm in *cog3* and to 282 nm in *cog8* (Fig 6J) as the resulted of increased widths of individual cisternae (Fig 6K). The overall changes in mini-stacks morphology were consistent with the changes in Golgi morphology revealed by GFP-EMP12 and GAUT14-GFP fluorescence (Fig 5). These observations suggested that COG3 and COG8 play a role in keeping normal Golgi morphology.

### Partial loss of the association of the COPI subunit γ-COP and its cargo protein EMP12 with the Golgi apparatus in *cog3* and *cog8* pollen

**Fig 7:**
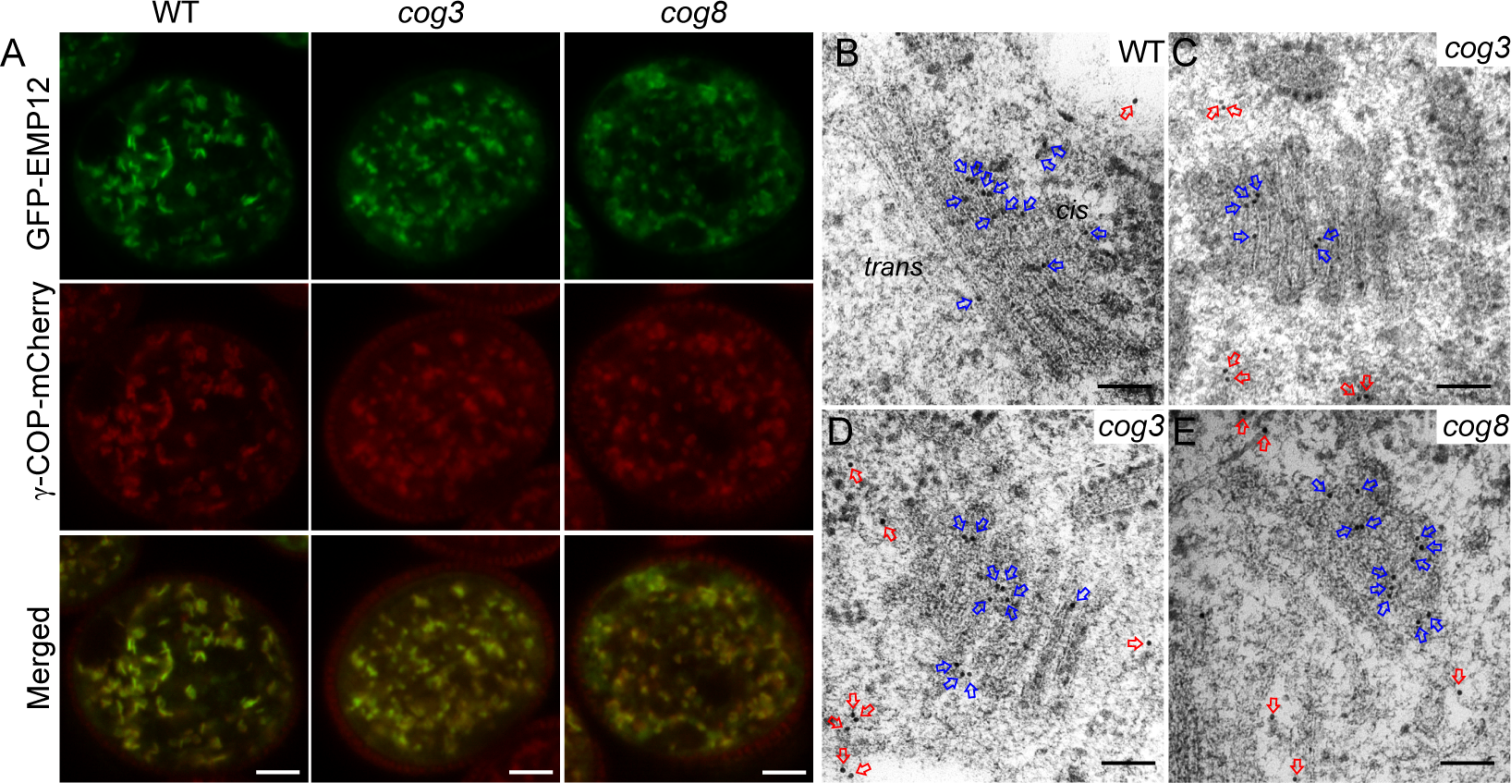
Disruption of γ-COP and EMP12 localization to the Golgi in *cog3* and *cog8* pollen. (**A**) γ-COP and EMP12 colocalized to the Golgi apparatus in wild-type pollen; however, γ-COP-mCherry and GFP-EMP12-labeled Golgi apparatus exhibited altered morphology, and the signals are more diffuse in the *cog3* and *cog8* pollen. (**B**) to (**E**) Ultra-thin sections cut from high-pressure frozen/freeze-substituted samples of wild-type (**B**), *cog3* (**C** and **D**), and *cog8* (**E**) were immunogold-labeled with EMP12 antibodies. The gold particles present in the cytoplasm (red arrows) and on the Golgi bodies (blue arrow) were shown. Note that EMP12 proteins (red arrows) appeared in the cytosol of *cog3* and *cog8* mutant pollen greatly outnumbered those in wild-type pollen. Bars = 5 μm in (**A**) and 100 nm in (**B**) to (**E**).

One possible mechanism by which the COG complex maintains Golgi integrity is the tethering of COPI vesicles, which appear at the periphery of Golgi cisternae [25, 26]. γ-COP is a subunit of the COPI coat, which has been shown to localize to the Golgi in plant cells [25]. We introduced *proLAT52*:*γ-COP-mCherry* and *proUBQ10*:*GFP-EMP12* constructs into wild-type, *cog3*^*-/+*^, and *cog8*^*-/+*^ backgrounds by crossing with plants harboring either of the constructs. In wild-type, double-labeled pollen, typical disc- or bar-like Golgi labeling was observed for both GFP-EMP12 and γ-COP-mCherry and the fluorescent signals merged well (Fig 7A, n = 43). In about half of *cog3*^-/+^ and *cog8*^-/+^ mutant pollen (32/68, *cog3*^*-/+*^; 28/59, *cog8*^*-/+*^), however, the Golgi labeled by GFP-EMP12 and γ-COP-mCherry exhibited altered morphology in a fuzzy background (Fig 7A). The diffuse GFP-EMP12 fluorescence was indicative of its disassociation from the Golgi apparatus, while γ-COP signals may represent free coatomer complexes in the cytosol (Fig 7A). The Golgi-resident protein EMP12 interacts via its C-terminal K*X*D/E motif with the COPI subunit γ-COP and thereby achieves steady state *cis-* and *medial-*Golgi localization by retrograde transport of COPI vesicles [44]. We performed immunogold electron microscopy analysis using anti-EMP12 antibodies on ultrathin sections prepared as described above. Consistent with the published data [44], the majority of anti-EMP12 antibody conjugated gold particles in wild-type pollen grains appeared at the *cis-* and *medial-*Golgi (Fig 7B, blue arrows). In *cog3* and *cog8* pollen grains, however, more gold particles (compare Fig 7B to Fig 7C–7E, red arrows) were found scattered in the cytoplasm distant from the mini-stacks. These data indicated that a large proportion of COPI vesicles containing EMP12 proteins lost their tight association with the Golgi and were released into the cytoplasm in *cog3* and *cog8* mutant pollen presumably due to disassembly of the COG complex containing COG3 and COG8 subunits.

### Cell wall materials and proteins required for pollen tube growth are deposited incorrectly in *cog3* and *cog8* pollen tubes

**Fig 8:**
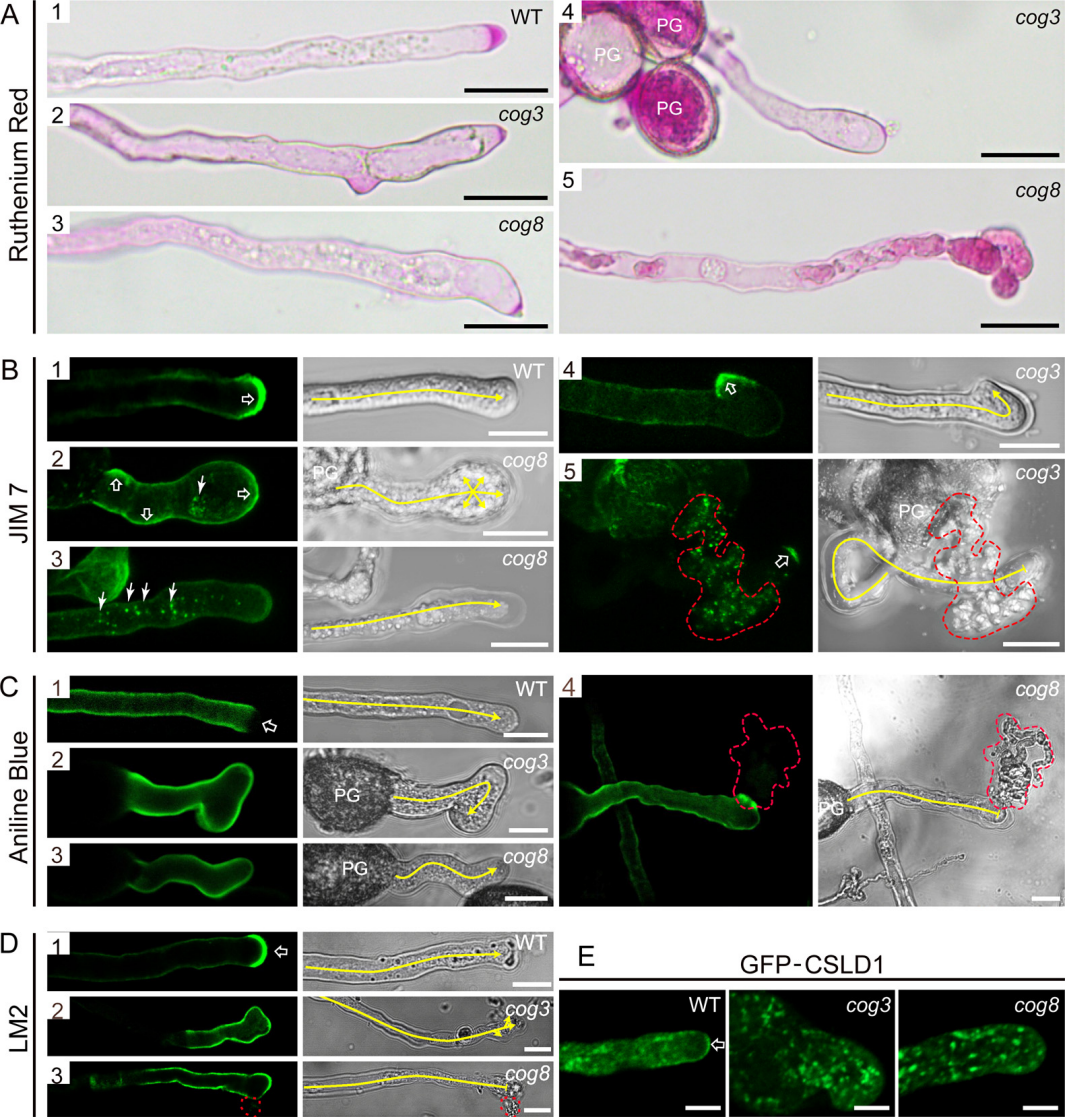
Altered distribution of cell wall materials and proteins in *cog3* and *cog8* pollen tubes. (**A**) Ruthenium red staining of pollen tubes. The crescent region in wild-type pollen tubes was stained heavily by ruthenium red (**A1**) and the deformed tips of *cog3* and *cog8* pollen tubes showed less staining (**A2**, **A3**, and **A4**). A bursting pollen tube with strong ruthenium red staining was trapped inside the cytosol (**A5**). (**B**) Confocal and corresponding DIC images of wild-type (**B1**), *cog3* (**B4**, **B5**), and *cog8* (**B2**, **B3**) pollen tubes labeled with JIM7 monoclonal antibody. Open arrow in (**B1**) indicates the crescent region of a wild-type pollen tube, while open arrows in (**B2**), (**B4**), and (**B5**) indicate the bulging tips of a curved pollen tube. Arrows in (**B2**) and (**B3**) indicate JIM7-positive structures inside *cog3* and *cog8* pollen tubes. (**C**) Confocal and corresponding DIC images of wild-type (**C1**), *cog3* (**C2**), or *cog8* (**C3**, **C4**) pollen tubes stained with aniline blue. Open arrow in (**C1**) indicates the tip region of a wild-type pollen tube. (**D**) Confocal and corresponding DIC images of wild-type (**D1**), *cog3* (**D2**), and *cog8* (**D3**) pollen tubes labeled with LM2 antibody. LM2 stained the very tip region (indicated by an open arrow) of wild-type pollen tubes. The tip-localization pattern of the LM2 signal was absent in *cog3* and *cog8* pollen tubes. (**E**) Confocal images of wild-type (**E1**), *cog3* (**E2**), and *cog8* (**E3**) pollen tubes expressing GFP-CSLD1. Red dashed lines in (**B5**), (**C4**), and (**D3**) highlight the cytoplasmic outflow of ruptured *cog3* and *cog8* pollen tubes. Yellow lines in **(B)**, **(C)**, and **(D)** indicate the pollen tube growth direction. Bars = 20 μm in (**A**); 10 μm in (**B**), (**C**), and (**D**); 5 μm in (**E**).

The *COG3* and *COG8* loss-of-function mutants exhibited restricted pollen tube growth (S3 Fig) and bulged, wavy, and ruptured pollen tubes (Fig 1), suggestive of altered deposition of cell wall components. Pectin is the major component of the pollen tube cell wall. Ruthenium red, which stains a broad range of methylesterified pectins, strongly labeled the tip region of wild-type pollen tubes (Fig 8A.1). In contrast, *cog3* and *cog8* pollen tubes exhibited lighter staining in confined regions in the deformed or branched pollen tube tips (Fig 8A.2–8A.4) and pectins appeared to accumulate in the cytosol when the pollen tubes were ruptured (Fig 8A.5). To examine the distribution of homogalacturonan (HG) with high and low degrees of methylesterification, we used the monoclonal antibodies JIM7 and JIM5, respectively [47]. The JIM7 antibody labeled the crescent of wild-type pollen tubes (Fig 8B.1) with a pattern similar to that previously reported for JIM7 labeling of pollen tubes [11, 48]. In some *cog3* and *cog8* pollen tubes, JIM7 signals were not tip-localized, but were instead distributed over the distal bulging tips (Fig 8B.2). Notably, JIM7-positive dots were present in intact (Fig 8B.2–8B.3) or ruptured (Fig 8B.5) pollen tubes indicating reduced secretion efficiency for JIM7-positive pectins. In addition, JIM7 signals were observed in a much smaller tip region at the curling tips (Fig 8B.4). In wild-type pollen tubes, JIM5 fluorescence was strong and uniform along the length of the pollen tube, except at the crescent (S7 Fig). In *cog3* and *cog8*, JIM5 labeling was essentially homogeneous along the entire pollen tube length (S7 Fig).

Callose is a component of the pollen tube inner wall. In wild-type pollen tubes, aniline blue-labeled callose was present along the flanks of the tube and was excluded from the pollen tube apex (Fig 8C.1). In the *cog3* and *cog8* mutants, callose was distributed evenly throughout the entire pollen tube wall (Fig 8C.2–8C.4). Based on these results, the distribution of some of the cell wall materials in the *cog3* and *cog8* mutants was altered; however, the pattern of cellulose was not affected (S7 Fig).

In addition to cell wall polysaccharides, we examined the localization of LM2 epitopes and GFP-CSLD1 fusion proteins. LM2 is a monoclonal antibody which recognizes a subset of arabinogalactan proteins (AGPs) [49]. LM2 labeling appeared more strongly at the tip than at the shank in the wild-type (Fig 8D.1). In *cog3* and *cog8* pollen tubes, the tip polarity of LM2 labeling was lost (Fig 8D.2 and 8D.3). CSLD1 is a putative cellulose synthase localizing to the Golgi apparatus that could be transported to the plasma membrane of pollen tube apex (Fig 8E) [43]. GFP-CSLD1 in *cog3* and *cog8* mutants retained the Golgi localization, but the apical membrane labeling was much decreased. Notably, the GFP-CSLD1 labeled Golgi bodies appeared in the clear zone where they were normally excluded (Fig 8E). Collectively, our data showed that in *cog3* and *cog8* mutants, polar deposition of cell wall materials and proteins is defective.

### Putative COG complex organization in plant cells

**Fig 9:**
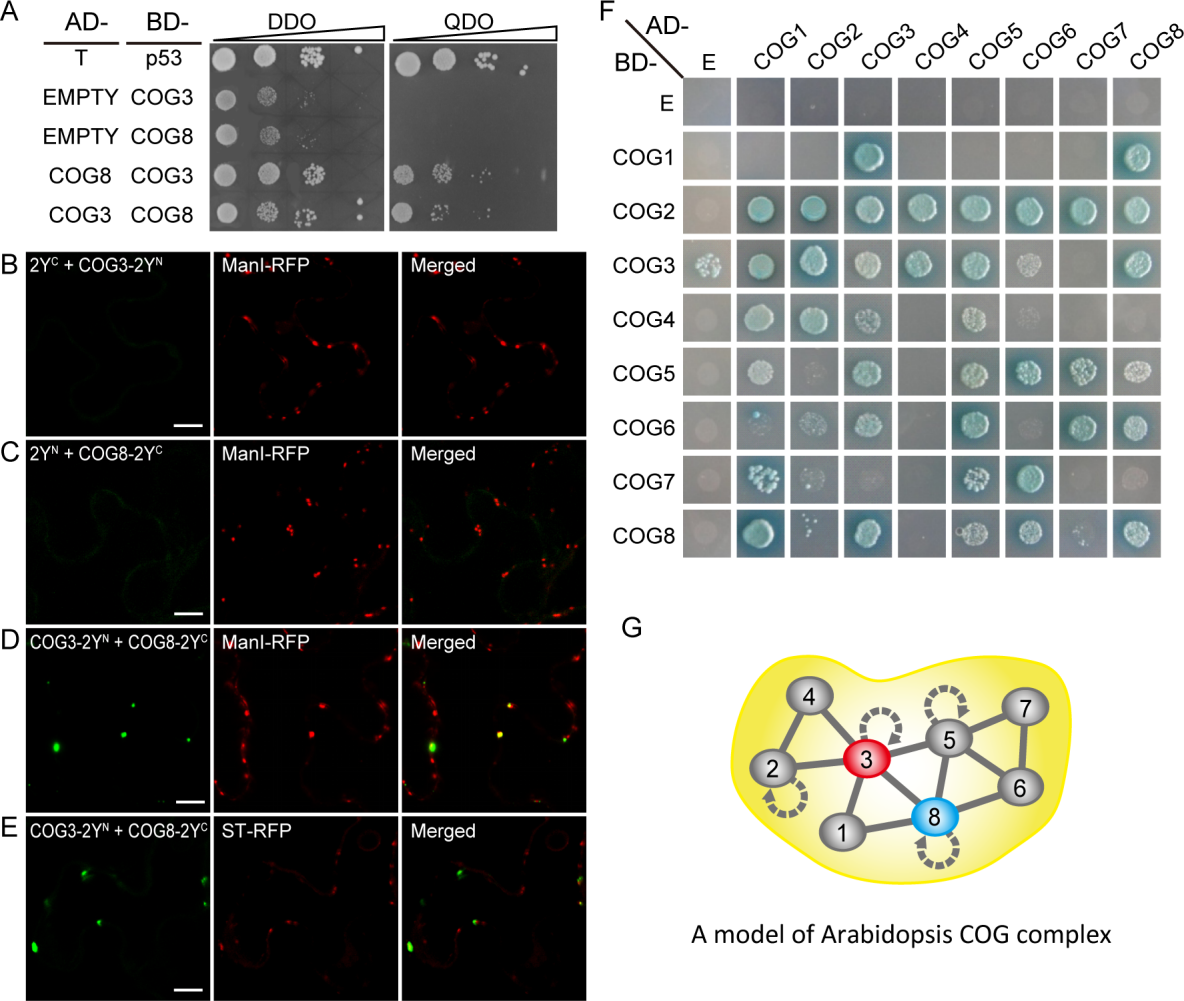
Interaction of COG3 and COG8 in a putative COG complex structural model. (**A**) Interaction of COG3 with COG8 in yeast. BD-p53 + AD-T served as a positive control. AD + COG3-BD and AD + COG8-BD served as negative controls. Growth on selective QDO plates indicated a positive interaction. (**B**) to (**E**) Interaction of COG3 and COG8 proteins in tobacco leaf cells as indicated by BIFC assay. Various combinations of plasmids are indicated. (**B**) and (**C**) Negative controls. (**D**) and (**E**) COG3 and COG8 interact at the Golgi apparatus. ManI-RFP: *cis*-Golgi marker, ST-RFP: *trans*-Golgi marker. (**F**) Representative result of pair-wise COG subunit interaction in yeast. E: empty vector. (**G**) The model of *Arabidopsis* COG complex organization.

The nearly identical phenotypes of the *cog3* and *cog8* mutants suggested that COG3 and COG8 may both be present in the COG complex and are critical for the function of the complex; therefore, we examined whether COG3 and COG8 interact directly and explored the structural organization of the COG complex. COG3 interacted with COG8 in yeast grown on a synthetic complete, quadruple drop out (QDO) medium lacking tryptophan, leucine, adenine, and histidine (Fig 9A). For bimolecular florescence complementation (BIFC) experiments, split-yellow fluorescent protein (YFP^N^ or YFP^C^) was fused to the C terminus of COG3 or COG8 respectively. COG3-2Y^N^ + 2Y^C^ and COG8-2Y^C^ + 2Y^N^ protein pairs served as negative controls (Fig 9B and 9C). Various combinations of plasmids were cotransfected into *Nicotiana benthamiana* leaves, and protein expression levels were examined (S8 Fig). YFP signals that localized to the Golgi were detected only with COG3-2Y^N^ and COG8-2Y^C^ cotransfection (Fig 9D and 9E), but not in controls (Fig 9B and 9C). To examine the architecture of the COG complex, we cloned eight subunits of the *Arabidopsis* COG complex into the pGADT7 and pGBKT7 vectors for pair wise bidirectional interaction tests using a yeast-two-hybrid assay. In the course of our study, we identified another gene in addition to *COG4* under the gene ID At4g01400, and based on our 3’ RACE data, *COG4* was given a new ID AT4G01395 by the Arabidopsis Information Resource (TAIR) (S9 Fig). Only those proteins that interacted bidirectionally were considered to reflect the COG complex structural assembly (Fig 9F). The analyses are summarized in Fig 9G. Interestingly, COG2, COG3, COG5 and COG8 were shown to have the abilities to form homodimers (Fig 9G, curved arrows). The interactions observed identified three possible COG complex subassemblies, COG2-4, COG1/8, and COG5-7, which appear to be well conserved in eukaryotic species.

## Discussion

Rapid tip growth of elongating pollen tubes is sustained by a highly dynamic intracellular trafficking system that targets transport vesicles to the apical clear zone. Our results show that the COG complex subunits COG3 and COG8 play important roles in Golgi structure maintenance, COPI vesicle tethering, and efficient anterograde Golgi trafficking, which is crucial for pollen tube stability, rapid tip-focused pollen tube growth, and male fertility.

### *cog3* and *cog8* pollen tube growth defects are caused by wrong deposition of cell wall materials

We screened T-DNA insertion mutants of several COG subunits and found that mutations in *COG3* and *COG8* caused similar defects in pollen tube growth. Consistently, COG3 and COG8 are co-expressed in mature pollen and pollen tube (Fig 1), and COG3 and COG8 interact directly (Fig 9). *cog3* and *cog8* mutant pollen tubes were burst, short and slow-growing, or wavy (Figs 1, S2 and S3), which resulted in male sterility (Table 1). Wrong deposition of cell wall materials could be the cause of various pollen tube defects. Highly methylesterified pectins which were labeled by the JIM7 antibody were absent from the pollen tube tip, but were instead distributed along the pollen tube wall which decreased the rigidity of the cell wall and caused the pollen tubes to rupture at random sites (S2 Fig). In contrast, JIM5-labeled demethylesterified pectins and calloses appeared at the pollen tube tip where they were normally absent, which restricted tip growth and resulted in short pollen tubes that did not grow efficiently (Figs 8 and S7). Various combinations of these incorrectly-deposited cell wall materials led to pleiotropic phenotypes including short and wavy, short and burst, and branched pollen tubes similar to phenotypes found in mutants in cell wall synthesis or modification [42, 45, 50, 51] or in vesicle trafficking pathways that deliver cell wall materials [11, 16]. The broad range of *cog3* and *cog8* phenotypes further highlights the importance of the COG complex in efficient secretory transport of cell wall materials which in turn is required to maintain the rapid growth rates. Also, we need to bear in mind that COG3 and COG8 are required for plant sporophytic development (Fig 3), and the detailed mechanisms await to be studied in the future.

### COG3 and COG8 are required for maintaining normal Golgi morphology

The Golgi apparatus consists of hundreds of individual stacks of flattened cisternae which are highly mobile in plant cells [31, 52]. Information on the nature of the molecular interactions that maintain the Golgi morphology is limited.

COG3 and COG8 localize exclusively in the Golgi apparatus, preferably at its periphery (Fig 4), resemble that of γ-COP localization [25]. In the *cog3* and *cog8* pollen, Golgi morphology were disrupted as evidenced by confocal microscopy (Fig 5) and TEM (Fig 6) analyses. Loss of COG3 and COG8 in the mutants may disassemble the entire COG complex, whereby COPI vesicles containing transmembrane protein EMP12 could not be tethered with the Golgi apparatus, and distributed to the cytosol (Fig 7). Therefore, we speculated that in the *cog3* and *cog8* pollen, the shorter cisternae were resulted from a loss of their rims by losing peripheral COPI vesicles. The Golgi-localized ARF-GEF GNOM-like 1 (GNL1) protein localizes to and acts mainly at the Golgi stacks [52]. Golgi cisternae were shown to be wider in the *gnl1* mutant probably due to the failure of COPI-coated vesicle formation [52, 53]. Thus, COPI vesicles located at the Golgi periphery seem to contribute to the maintenance of Golgi morphology in plant cells.

In mammalian cells, the two characteristic features of the Golgi apparatus, flattened cisternae and cisternal stacking, is proposed to be governed by Golgi cisternal adhesion [54]. Major players of cisternal adhesion in mammalian cells, such as GRASP65/55, Glogin45 and GM130, are either not identified or not functioning in Golgi stacking in plant [54]. Instead, multiple weak inter cisternal adhesive processes (e.g., Rab-SNARE, tether-SNARE) could be underlying the plant Golgi morphology maintenance [54]. In the *cog3* and *cog8* pollen, the widths of individual Golgi cisternae increased about 40% in the mini-stacks (Fig 6K), indicative of disruption in cisternal flatness, and COG3 and COG8 as one of the players of intercisternal adhesion. In mammalian cells, the COG complex might both physically and functionally interacts with Rabs, SNAREs, SNARE-interacting proteins, coiled-coil tethers (golgin 84, p115, CASP, TMF etc.) [23]. If COG3 and COG8 proteins interact with these components in plant cells, *cog3* and *cog8* mutations might lead to the un-stability of intercisternal holding. However, these possibilities awaits further studies.

### Aberrant Golgi membranes support anterograde transport at a lower rate and lower accuracy

The Golgi serves as a station for protein sorting and transport, receiving membrane and proteins from the ER and delivering them to the plasma membrane or other intracellular sites. In the meantime, Golgi-resident membrane proteins are thought to undergo continuous cycling between the ER and Golgi in plant cells [17, 32]. In the *cog3* and *cog8* mutants, two Golgi-resident membrane proteins EMP12 and GAUT14 (Fig 5) were able to localize to the aberrant Golgi bodies, but not redistributed to the ER, indicating that protein exchange between the ER and the Golgi apparatus was not interrupted. What happened to the vesicle trafficking from the Golgi to the plasma membrane in the two mutant pollen tubes? Pectins and structural cell wall proteins are synthesized and modified within the Golgi apparatus after which they are packaged into secretory vesicles for targeting to sites of new cell wall deposition [51]. The disrupted Golgi bodies were shown to support anterograde delivery from the Golgi to the plasma membrane (Fig 8), but with much lower efficiency than the normal ones, as indicated by the presence of JIM7 fluorescence dots in the cytoplasm (Fig 8B), and by GFP-CSLD1 proteins less efficiently delivered to the apex pollen tube membrane (Fig 8E). Similar results were obtained in mammalian cells in which the anterograde transport of vesicular stomatitis virus G protein (VSVG) through the Golgi had minor defects and/or kinetic delays by partial COG3 depletion [21]. Furthermore, cell wall components examined in Fig 8 all exhibited wrong localization in the mutant pollen tubes. Therefore, COG complex is required for highly efficient and accurate anterograde transport.

### *Arabidopsis* COG complex organization maybe similar to that in yeast and mammals

The direct interaction of COG3 and COG8 indicated by the yeast-two-hybrid (Fig 9A) and BIFC (Fig 9B–9E) assays is not surprising given the similar defects in pollen tube growth caused by mutations in *COG3* and *COG8* (Fig 1). The *cog3* and *cog8* phenotypes are more severe than those of *cog7* [37] probably because COG3 and COG8 have more interacting partners within the putative *Arabidopsis* COG complex (Fig 9F and 9G) and disruption of COG3 or COG8 likely results in disassembly of the entire COG complex. In mammalian cells, the eight subunits that make up the COG complex can be divided into three sub-assemblies: COG2-4, COG5-7, and COG1/8. The COG1/8 subassembly bridges the COG2-4 and COG5-7 assemblies, while, in the yeast COG complex, COG1p links the COG2-4p and COG5-7p lobes [20]. Our proposed model of the *Arabidopsis* COG complex organization largely resembles those of yeast and mammals, but with clear differences. For example, AtCOG8 appears to link the AtCOG2-4p and AtCOG5-7p lobes and certain pairs of subunit interactions are specific to plants. But the proposed model needs supports from further experiments, and the functional significance of the model awaits to be explored.

In this study, we show that the functions of COG complex are coupled to pollen tube growth, and provide important insights into the structure maintenance and the functioning of the Golgi apparatus in plant cells.

## Materials and Methods

### Plant materials and growth conditions

All *Arabidopsis* plants used in this study are of the Col-0 ecotype. The T-DNA insertion mutants *cog3*^*-*/+^ (GK_498G10) and *cog8*^*-*/+^ (SALK_122096) were obtained from the *Arabidopsis* Information Resource (TAIR, www.arabidopsis.org). GAUT14-GFP, GFP-CSLD1, and RPA-DsRed transgenic lines were kindly provided by Dr. Liqun Chen [45], Dr. De Ye [43], and Dr. Weicai Yang [42], respectively. Plants were grown at 22°C under a cycle of 16 h light/8 h dark.

### Phenotype characterization

To visualize nuclei, pollen grains were collected into a 4′,6-diamidino-2-phenylindole (DAPI) staining solution (0.1 M sodium phosphate pH 7.0, 1 mM EDTA, 0.1% Triton X-100, and 0.5 mg/mL DAPI) and examined 15 min after staining. Pollen viability was examined using Alexander staining [55]. To observe embryo development, developing seeds were clarified overnight in Hoyer’s solution [56] and observed with a Zeiss microscope equipped with a Differential Interference Contrast (DIC) system. *In vitro* pollen germination was performed as described by [51]. Germinating pollen grains were counted under a microscope after incubation at 23°C for 6 h.

### Confocal laser scanning microscopy

Confocal images of fluorescent proteins or immuno-fluorescence signals in pollen grains or pollen tubes were collected using an LSM710 system (Zeiss, www.zeiss.com). Green fluorescent protein (GFP) signals were excited at 488 nm and emission was detected at 505–530 nm. RPA-DsRed [42] signals were excited at 543 nm and emission was detected at 585–615 nm. Time-lapse movies and images were obtained using an Ultra View spinning-disc confocal scanner unit (PerkinElmer) and processed using Velocity software (5.3V, Improvision, Inc.).

### Immunofluorescence and cytochemical staining

Pollen tubes were adhered to poly-L-lysine-covered glass slides after germination in liquid germination medium for 4 h. Pollen tubes were fixed in 4% (w/v) polyformaldehyde in PIPES buffer (50 mM PIPES, 1 mM EGTA, 5 mM MgSO_4_, 0.5 mM CaCl_2_, 0.1% TritonX-100, pH 7) for 1 h. The fixed pollen tubes were washed three times with PBS (100 mM potassium phosphate, 138 mM NaCl, and 2.7 mM KCl, pH 7.3) and then incubated in blocking buffer (0.8% BSA, 0.1% gelatin, and 2 mM NaN_3_ in PBS) for 30 min. After removing the blocking buffer, pollen tubes were treated with primary antibody for 1 h. The carbohydrate antibodies JIM5, JIM7, and LM2 were diluted 1:100 in blocking buffer. The pollen tubes were washed three times with PBS and incubated for 30 min in Alexa488 conjugated secondary antibody (1:100 dilution in blocking buffer). The pollen tubes were washed five times with PBS before analysis using a Zeiss LSM710 confocal microscope. For cytochemical staining, the pollen tubes were stained without fixation after germination in liquid germination medium for 4 h. Calcofluor white (0.01%, w/v), aniline blue (0.1%, w/v), and ruthenium red (0.01%, w/v) in germination medium were used to stain β-glucans (both cellulose and callose), callose, and pectins, respectively in pollen tubes.

### Scanning and transmission electron microscopy

For scanning electron microscopy (SEM) observation, pollen grains were mounted on sample stubs. After dehydration in air for 30 min, pollen grains were coated with gold particles (EIKO IB-3). Pollen grains were then observed using a HITACHI S-3000N scanning electron microscope.

Immunogold labeling were performed essentially as described previously [44]. Anthers of *qrt1*^-/-^, *cog3*^-/+^ *qrt1*^-/-^, and *cog8*^-/+^ *qrt1*^-/-^ flowers were cut and immediately frozen in a high-pressure freezer (EM PACT2; Leica) followed by subsequent freeze substitution in dry acetone containing 0.1% uranyl acetate at –85°C in an AFS freeze substitution unit (Leica). Infiltration with HM20, embedding, and ultraviolet (UV)-polymerization were performed stepwise at –35°C. Immunogold labeling was performed with GFP (80 mg/mL) or EMP12 antibodies (40 mg/mL) and gold-coupled secondary antibody at a 1:50 dilution.

To visualize the structure of Golgi stacks by TEM, anthers of *qrt1*^-/-^, *cog3*^-/+^ *qrt1*^-/-^, and *cog8*^-/+^ *qrt1*^-/-^ flowers were cut and high-pressure frozen and substituted with 2% OsO4 in 100% acetone and infiltrated with Epon resin as described [57]. TEM examination was performed using a Hitachi H-7650 transmission electron microscope with a charge-coupled device camera (Hitachi High-Technologies) operating at 80 kV.

### Bimolecular fluorescence complementation (BIFC) assay

The coding sequences of the *Arabidopsis* COG3 and COG8 genes were subcloned into the p2YN-35S and p2YC-35S vectors, respectively, which containing yellow fluorescent protein (YFP) fragments and HA tag at C terminal, resulting in COG3-YFP^N^ and COG8-YFP^C^ constructs. For transient expression, *Agrobacterium tumefaciens* cells carrying the BIFC constructs were coinfiltrated with constructs carrying the Golgi marker ManI-mCherry or ST-mCherry into five- to six-week-old *Nicotiana benthamiana* tobacco leaves. Infiltrated leaves were observed after 24 h. The eYFP and mCherry fluorescent signals were monitored sequentially. The excitation and detection wavelengths for eYFP and mCherry were 514 and 587 nm for excitation, 527 and 610 nm for detection, respectively. For western blot, the infiltrated leaves were ground in liquid nitrogen and extracted with the lysis buffer, following the SDS-PAGE and immunoblotting. Anti-HA antibodies (Abcam ab9110) were used as the primary antibody at a dilution of 1:500.

### Yeast two-hybrid assay

Yeast-two hybrid analysis was performed using the Match Maker GAL4 Two-Hybrid System according to the supplier’s instructions (Clontech). The cDNAs were cloned into the pGBKT7 and pGADT7 vectors (Clontech). Pairs of pGBKT7 and pGADT7 vectors were cotransformed into the *Saccharomyces cerevisiae* strain AH109. Diploids were selected on synthetic drop-out (SD) medium lacking Trp and Leu (SD-Trp-Leu, DDO), while the selection of yeast cells expressing interacting proteins was made on SD medium lacking Ade, His, Trp, and Leu (SD-Ade-His-Trp-Leu, QDO), and subjected to a β-galactosidase assay. The experiments (yeast transformation, mating, and selection) were repeated several times and the representative result was presented.

### Accession numbers

The AGI identifiers for the *Arabidopsis* genes described in this work are: *COG1*, At5g16300; *COG2*, At4g24840; *COG3*, AT1G73430; *COG4*, At4g01395; *COG5*, At1g67930; *COG6*, At1g31780; *COG7*, *At5g51430*; *COG8*, AT5G11980; *EMP12*, At1g10950; *GAUT14*, At5g15470; *CSLD1*, At2g33100; *RPA*, At2g35210; *γ-COP*, At4g34450.

## Supporting Information

S1 Fig *cog3* and *cog8* pollen grains are morphologically and developmentally normal. (**A**) *qrt1*^*-/-*^, *cog3*^*-/+*^ *qrt1*^*-/-*^, and *cog8*^*-/+*^ *qrt1*^*-/-*^ quartets exhibit similar Alexander staining patterns. (**B**) *qrt1*^*-/-*^, *cog3*^*-/+*^ *qrt1*^*-/-*^, and *cog8*^*-/+*^ *qrt1*^*-/-*^ quartets are normal in appearance as revealed by scanning electron microscopy (SEM) observation. (**C**) DAPI staining showing that each mature pollen grain of *qrt1*^*-/-*^, *cog3*^*-/+*^ *qrt1*^*-/-*^, and *cog8*^*-/+*^ *qrt1*^*-/-*^ quartets contains two sperm cells and one vegetative cell. Bars = 10 μm in (**A**), (**B**), and (**C**).
(TIF)

S2 Fig *In vitro* growth of pollen tubes of *cog3*^*-/+*^ and *cog8*^*-/+*^ heterozygous mutants. (**A**), (**B**), and (**C**) *In vitro* growth of wild-type, *cog3*^*-/+*^, and *cog8*^*-/+*^ pollen tubes, respectively. (**D**) *In vitro* growth of *proLAT52*:*COG3-GFP* complemented *cog3*^*-/+*^ (*cog3*^*-/+*^ *proLAT52*:*COG3-GFP/proLAT52*:*COG3-GFP*) pollen tubes. (**E**) *In vitro* growth of *proLAT52*:*COG8-GFP* complemented *cog8*^*-/+*^ (*cog8*^*-/+*^ *proLAT52*:*COG3-GFP/proLAT52*:*COG8-GFP*) pollen tubes. (**F**) and (**G**) Burst pollen tubes in *cog3*^*-/+*^ and *cog8*^*-/+*^ mutants, respectively. (**H**) *In vitro* growth of wild-type pollen tube. (**I**) to (**L**) Pleiotropic phenotypes of *cog3*^*-/+*^ and *cog8*^*-/+*^mutant pollen tubes. Short and swollen (**I**), wavy (**J**), burst (**K**), and branched (**L**) pollen tubes. (**M**) Statistical analysis of various phenotypes in wild-type, *cog3*^*-/+*^, *cog8*^*-/+*^, *proLAT52*:*COG3-GFP* complemented *cog3*^*-/+*^, and *proLAT52*:*COG8-GFP* complemented *cog8*^*-/+*^ pollen tubes. n> 500 for each genotype, values represent the means ± SD. Bars = 50 μm in (**A**) to (**C**); 20 μm in (**D**) to (**L**).
(TIF)

S3 Fig *cog3* and *cog8* pollen tubes exhibited stunted growth. (**A**) Time-lapse images of wild-type, *cog3*, and *cog8* pollen tubes. (**B**) Growth of *cog3* and *cog8* pollen tubes was significantly slower than that of wild-type tubes. Six pollen tubes each of wild-type, *cog3*, and *cog8* were measured. Values represent the means ± SD. **P< 0.001 by Student’s *t* test. Bars = 20 μm.
(TIF)

S4 Fig *COG3* and *COG8* genes are expressed ubiquitously. (**A**) Quantitative RT-PCR assays of *COG3* and *COG8* genes showed that they are expressed in all tissues examined with the highest expression levels in siliques. (**B**, **D**, **F**, **H**, **J**) *proCOG3*:*GUS* expression pattern. *proCOG3*:*GUS* is expressed in embryos (**B**), young seedlings (**D**), seedlings with cauline leaves (**F**), anthers (**H**), and mature pollen (**J**). (**C**, **E**, **G**, **I**, **K**) *proCOG8*:*GUS* expression pattern in the same tissues examined in *proCOG3*:*GUS* plants. Bars = 0.1 mm in (**B** and **C**), 1 mm in (**D**–**G**), 0.5 mm in (**H** and **I**), and 10 μm in (**J** and **K**).
(TIF)

S5 Fig *COG8* genomic DNA rescued the phenotypes of *cog8* mutants. (**A**) *COG8* gene structure and primers used for genotyping. (**B**) Genotyping of a representative line of *COG8* genomic DNA transformed *cog8* mutants, which showed hygromycin resistance. Note that the primer1305-P1 located on the vector is not shown here. (**C**) *cog8*^-/-^ *gCOG8*/*gCOG8* plants grow normally. (**D**) Pollen tubes from *cog8*^-/-^ *gCOG8*/*gCOG8* plants grow normally. Bars = 10 cm in (C), 50 μm in (D).
(TIF)

S6 Fig COG3 and COG8 proteins are not localized to the ER. COG3-GFP and COG8-GFP signals are not overlapped with the ER marker mCherry-HDEL in the pollen grains and the pollen tubes. Bars = 10 μm.
(TIF)

S7 Fig Calcofluor white and JIM5 labeling of *cog3* and *cog8* pollen tubes. (**A**) Approximately homogeneous distribution of Calcofluor white staining in the cell wall of a wild-type pollen tube (**A1**), and similar staining patterns in deformed *cog3* and *cog8* pollen tubes (**A2** and **A3**). (**B**) JIM5 epitopes were absent at the tip of a wild-type pollen tube (**B1**), but present at the tips of *cog3* and *cog8* mutant pollen tubes (**B2** and **B3**). Yellow lines indicate pollen tube growth directions. Red dashed lines in (**A3**) highlight the cytoplastic outflow of ruptured *cog3*and *cog8* pollen tubes. Bars = 10 μm.
(TIF)

S8 Fig Various fusion proteins in samples for BIFC assay were expressed properly. (TIF)

S9 Fig Re-annotation of the *COG4* gene. Based on our 3’ RACE data, TAIR assigned a new accession number (At4g01395) to *COG4* gene. (TIF)

S1 Table Complementation analysis of *cog8*^*-/+*^ *gCOG8*^*-/+*^ lines. (DOC)

S2 Table Characterization of the progeny of *cog3*^*-/+*^ *proLAT52*:*COG3-GFP/proLAT52*:*COG3-GFP* and *cog8*^*-/+*^ *proLAT52*:*COG8-GFP/proLAT52*:*COG8-GFP* mutants. (DOC)

S3 Table List of primer pairs used in this study. (DOC)

S1 Movie Live-cell imaging of COG3-GFP in wild-type pollen tubes. (MOV)

S2 Movie Live-cell imaging of COG8-GFP in wild-type pollen tubes. (MOV)

## References

[1] McCormick S (2004) Control of male gametophyte development. Plant Cell 16 Suppl: S142–153. 15037731 10.1105/tpc.016659 PMC2643393

[2] Hepler PK, Vidali L, Cheung AY (2001) Polarized cell growth in higher plants. Annu Rev Cell Dev Biol 17: 159–187. 11687487 10.1146/annurev.cellbio.17.1.159

[3] Chebli Y, Kroeger J, Geitmann A (2013) Transport logistics in pollen tubes. Mol Plant 6: 1037–1052. 10.1093/mp/sst073 23686949

[4] Rounds CM, Bezanilla M (2013) Growth mechanisms in tip-growing plant cells. Annu Rev Plant Biol 64: 243–265. 10.1146/annurev-arplant-050312-120150 23451782

[5] Derksen J, Rutten T, Lichtscheidl IK, Win AHND, Pierson ES, et al (1995) Quantitative analysis of the distribution of organelles in tobacco pollen tubes: implications for exocytosis and endocytosis. Protoplasma 188: 267–276.

[6] Moscatelli A, Ciampolini F, Rodighiero S, Onelli E, Cresti M, et al (2007) Distinct endocytic pathways identified in tobacco pollen tubes using charged nanogold. J Cell Sci 120: 3804–3819. 17940063 10.1242/jcs.012138

[7] Bove J, Vaillancourt B, Kroeger J, Hepler PK, Wiseman PW, et al (2008) Magnitude and direction of vesicle dynamics in growing pollen tubes using spatiotemporal image correlation spectroscopy and fluorescence recovery after photobleaching. Plant Physiol 147: 1646–1658. 10.1104/pp.108.120212 18508956 PMC2492615

[8] Zonia L, Munnik T (2008) Vesicle trafficking dynamics and visualization of zones of exocytosis and endocytosis in tobacco pollen tubes. J Exp Bot 59: 861–873. 10.1093/jxb/ern007 18304978

[9] Cheung AY, Wu HM (2008) Structural and signaling networks for the polar cell growth machinery in pollen tubes. Annu Rev Plant Biol 59: 547–572. 10.1146/annurev.arplant.59.032607.092921 18444907

[10] de Graaf BH, Cheung AY, Andreyeva T, Levasseur K, Kieliszewski M, et al (2005) Rab11 GTPase-regulated membrane trafficking is crucial for tip-focused pollen tube growth in tobacco. Plant Cell 17: 2564–2579. 16100336 10.1105/tpc.105.033183 PMC1197435

[11] Szumlanski AL, Nielsen E (2009) The Rab GTPase RabA4d regulates pollen tube tip growth in *Arabidopsis thaliana*. Plant Cell 21: 526–544. 10.1105/tpc.108.060277 19208902 PMC2660625

[12] Hala M, Cole R, Synek L, Drdova E, Pecenkova T, et al (2008) An Exocyst complex functions in plant cell growth in *Arabidopsis* and Tobacco. Plant Cell 20: 1330–1345. 10.1105/tpc.108.059105 18492870 PMC2438459

[13] Drdova EJ, Synek L, Pecenkova T, Hala M, Kulich I, et al (2013) The exocyst complex contributes to PIN auxin efflux carrier recycling and polar auxin transport in *Arabidopsis*. Plant J 73: 709–719. 10.1111/tpj.12074 23163883

[14] Wu J, Tan X, Wu C, Cao K, Li Y, et al (2013) Regulation of cytokinesis by exocyst subunit SEC6 and KEULE in *Arabidopsis thaliana*. Mol Plant 6: 1863–1876. 10.1093/mp/sst082 23702595

[15] Richter S, Muller LM, Stierhof YD, Mayer U, Takada N, et al (2012) Polarized cell growth in *Arabidopsis* requires endosomal recycling mediated by GBF1-related ARF exchange factors. Nat Cell Biol 14: 80–86. 10.1038/ncb2389 22138577

[16] Cheung AY, Chen CYH, Glaven RH, de Graaf BHJ, Vidali L, et al (2002) Rab2 GTPase regulates vesicle trafficking between the endoplasmic reticulum and the Golgi bodies and is important to pollen tube growth. Plant Cell 14: 945–962. 11971147 10.1105/tpc.000836 PMC150694

[17] Stefano G, Renna L, Chatre L, Hanton SL, Moreau P, et al (2006) In tobacco leaf epidermal cells, the integrity of protein export from the endoplasmic reticulum and of ER export sites depends on active COPI machinery. Plant J 46: 95–110. 16553898 10.1111/j.1365-313X.2006.02675.x

[18] Ito Y, Uemura T, Nakano A (2014) Formation and maintenance of the Golgi apparatus in plant cells. Int Rev Cell Mol Biol 310: 221–287. 10.1016/B978-0-12-800180-6.00006-2 24725428

[19] Ram RJ, Li B, Kaiser CA (2002) Identification of Sec36p, Sec37p, and Sec38p: components of yeast complex that contains Sec34p and Sec35p. Mol Biol Cell 13: 1484–1500. 12006647 10.1091/mbc.01-10-0495 PMC111121

[20] Ungar D, Oka T, Krieger M, Hughson FM (2006) Retrograde transport on the COG railway. Trends in Cell Biology 16: 113–120. 16406524 10.1016/j.tcb.2005.12.004

[21] Zolov SN, Lupashin VV (2005) Cog3p depletion blocks vesicle-mediated Golgi retrograde trafficking in HeLa cells. J Cell Biol 168: 747–759. 15728195 10.1083/jcb.200412003 PMC2171815

[22] Sohda M, Misumi Y, Yamamoto A, Nakamura N, Ogata S, et al (2010) Interaction of Golgin-84 with the COG complex mediates the intra-Golgi retrograde transport. Traffic 11: 1552–1566. 10.1111/j.1600-0854.2010.01123.x 20874812

[23] Willett R, Ungar D, Lupashin V (2013) The Golgi puppet master: COG complex at center stage of membrane trafficking interactions. Histochem Cell Biol 140: 271–283. 10.1007/s00418-013-1117-6 23839779 PMC3748202

[24] Latijnhouwers M, Hawes C, Carvalho C (2005) Holding it all together? Candidate proteins for the plant Golgi matrix. Curr Opin Plant Biol 8: 632–639. 16194619 10.1016/j.pbi.2005.09.014

[25] Pimpl P, Movafeghi A, Coughlan S, Denecke J, Hillmer S, et al (2000) In situ localization and in vitro induction of plant COPI-coated vesicles. Plant Cell 12: 2219–2236. 11090220 10.1105/tpc.12.11.2219 PMC150169

[26] Donohoe BS, Kang BH, Staehelin LA (2007) Identification and characterization of COPIa- and COPIb-type vesicle classes associated with plant and algal Golgi. Proc Natl Acad Sci U S A 104: 163–168. 17185411 10.1073/pnas.0609818104 PMC1765428

[27] Contreras I, Ortiz-Zapater E, Castilho LM, Aniento F (2000) Characterization of Cop I coat proteins in plant cells. Biochem Biophys Res Commun 273: 176–182. 10873582 10.1006/bbrc.2000.2918

[28] Couchy I, Bolte S, Crosnier MT, Brown S, Satiat-Jeunemaitre B (2003) Identification and localization of a beta-COP-like protein involved in the morphodynamics of the plant Golgi apparatus. J Exp Bot 54: 2053–2063. 12885863 10.1093/jxb/erg230

[29] Takeuchi M, Ueda T, Yahara N, Nakano A (2002) Arf1 GTPase plays roles in the protein traffic between the endoplasmic reticulum and the Golgi apparatus in tobacco and *Arabidopsis* cultured cells. Plant J 31: 499–515. 12182707 10.1046/j.1365-313x.2002.01372.x

[30] Matheson LA, Hanton SL, Rossi M, Latijnhouwers M, Stefano G, et al (2007) Multiple roles of ADP-ribosylation factor 1 in plant cells include spatially regulated recruitment of coatomer and elements of the Golgi matrix. Plant Physiol 143: 1615–1627. 17307898 10.1104/pp.106.094953 PMC1851833

[31] Boevink P, Oparka K, Santa Cruz S, Martin B, Betteridge A, et al (1998) Stacks on tracks: the plant Golgi apparatus traffics on an actin/ER network. Plant J 15: 441–447. 9750355 10.1046/j.1365-313x.1998.00208.x

[32] Brandizzi F, Snapp EL, Roberts AG, Lippincott-Schwartz J, Hawes C (2002) Membrane protein transport between the endoplasmic reticulum and the Golgi in tobacco leaves is energy dependent but cytoskeleton independent: evidence from selective photo bleaching. Plant Cell 14: 1293–1309. 12084828 10.1105/tpc.001586 PMC150781

[33] Ramirez IB, Lowe M (2009) Golgins and GRASPs: holding the Golgi together. Semin Cell Dev Biol 20: 770–779. 10.1016/j.semcdb.2009.03.011 19508854

[34] Renna L, Hanton SL, Stefano G, Bortolotti L, Misra V, et al (2005) Identification and characterization of AtCASP, a plant transmembrane Golgi matrix protein. Plant Mol Biol 58: 109–122. 16028120 10.1007/s11103-005-4618-4

[35] Latijnhouwers M, Gillespie T, Boevink P, Kriechbaumer V, Hawes C, et al (2007) Localization and domain characterization of *Arabidopsis* golgin candidates. J Exp Bot 58: 4373–4386. 10.1093/jxb/erm304 18182439

[36] Kang BH, Staehelin LA (2008) ER-to-Golgi transport by COPII vesicles in *Arabidopsis* involves a ribosome-excluding scaffold that is transferred with the vesicles to the Golgi matrix. Protoplasma 234: 51–64. 10.1007/s00709-008-0015-6 18810574

[37] Ishikawa T, Machida C, Yoshioka Y, Ueda T, Nakano A, et al (2008) *EMBRYO YELLOW* gene, encoding a subunit of the conserved oligomeric Golgi complex, is required for appropriate cell expansion and meristem organization in *Arabidopsis thaliana*. Genes Cells 13: 521–535. 10.1111/j.1365-2443.2008.01186.x 18422605

[38] Ostertag M, Stammler J, Douchkov D, Eichmann R, Huckelhoven R (2013) The conserved oligomeric Golgi complex is involved in penetration resistance of barley to the barley powdery mildew fungus. Mol Plant Pathol 14: 230–240. 10.1111/j.1364-3703.2012.00846.x 23145810 PMC6638642

[39] Preuss D, Rhee SY, Davis RW (1994) Tetrad analysis possible in *Arabidopsis* with mutation of the *QUARTET* (*QRT*) genes. Science 264: 1458–1460. 8197459 10.1126/science.8197459

[40] Zinkl GM, Zwiebel BI, Grier DG, Preuss D (1999) Pollen-stigma adhesion in *Arabidopsis*: a species-specific interaction mediated by lipophilic molecules in the pollen exine. Development 126: 5431–5440. 10556067 10.1242/dev.126.23.5431

[41] Song X-F, Yang C-Y, Liu J, Yang W-C (2006) RPA, a class II ARFGAP Protein, activates ARF1 and U5 and plays a role in root hair development in *Arabidopsis*. Plant Physiology 141: 966–976. 16731582 10.1104/pp.106.077818 PMC1489917

[42] Wang W, Wang L, Chen C, Xiong G, Tan XY, et al (2011) *Arabidopsis* CSLD1 and CSLD4 are required for cellulose deposition and normal growth of pollen tubes. J Exp Bot 62: 5161–5177. 10.1093/jxb/err221 21765162 PMC3193019

[43] Crofts A.J., Leborgne-Castel N., Hillmer S., Robinson D.G., Phillipson B., Carlsson L.E., et al (1999) Saturation of the endoplasmic reticulum retention machinery reveals anterograde bulk flow. Plant Cell 11: 2233–2248. 10559446 10.1105/tpc.11.11.2233 PMC144130

[44] Gao C, Yu CK, Qu S, San MW, Li KY, et al (2012) The Golgi-localized *Arabidopsis* endomembrane protein12 contains both endoplasmic reticulum export and Golgi retention signals at its C terminus. Plant Cell 24: 2086–2104. 10.1105/tpc.112.096057 22570441 PMC3442589

[45] Wang L, Wang W, Wang YQ, Liu YY, Wang JX, et al (2013) *Arabidopsis* galacturonosyltransferase (GAUT) 13 and GAUT14 have redundant functions in pollen tube growth. Mol Plant 6: 1131–1148. 10.1093/mp/sst084 23709340

[46] Nebenfuhr A, Gallagher LA, Dunahay TG, Frohlick JA, Mazurkiewicz AM, et al (1999) Stop-and-go movements of plant Golgi stacks are mediated by the acto-myosin system. Plant Physiol 121: 1127–1142. 10594100 10.1104/pp.121.4.1127 PMC59480

[47] Chebli Y, Kaneda M, Zerzour R, Geitmann A (2012) The cell wall of the *Arabidopsis* pollen tube—spatial distribution, recycling, and network formation of polysaccharides. Plant Physiol 160: 1940–1955. 10.1104/pp.112.199729 23037507 PMC3510122

[48] Bosch M, Hepler PK (2005) Pectin methylesterases and pectin dynamics in pollen tubes. Plant Cell 17: 3219–3226. 16322606 10.1105/tpc.105.037473 PMC1315365

[49] Dardelle F, Lehner A, Ramdani Y, Bardor M, Lerouge P, et al (2010) Biochemical and immunocytological characterizations of *Arabidopsis* pollen tube cell wall. Plant Physiol 153: 1563–1576. 10.1104/pp.110.158881 20547702 PMC2923879

[50] Jiang L, Yang SL, Xie LF, Puah CS, Zhang XQ, et al (2005) *VANGUARD1* encodes a pectin methylesterase that enhances pollen tube growth in the *Arabidopsis* style and transmitting tract. Plant Cell 17: 584–596. 15659637 10.1105/tpc.104.027631 PMC548828

[51] Gu F, Nielsen E (2013) Targeting and regulation of cell wall synthesis during tip growth in plants. J Integr Plant Biol 55: 835–846. 10.1111/jipb.12077 23758901

[52] Staehelin LA, Moore I (1995) The plant Golgi apparatus: Structure, functional organization and trafficking mechanisms. Annu Rev Plant Physiol Plant MolBiol 46: 261–288.

[53] Richter S, Geldner N, Schrader J, Wolters H, Stierhof YD, et al (2007) Functional diversification of closely related ARF-GEFs in protein secretion and recycling. Nature 448: 488–492. 17653190 10.1038/nature05967

[54] Teh OK, Moore I (2007) An ARF-GEF acting at the Golgi and in selective endocytosis in polarized plant cells. Nature 448: 493–496. 17653191 10.1038/nature06023

[55] Alexander MP (1969) Differential staining of aborted and nonaborted pollen. Stain Technol 44: 117–122. 4181665 10.3109/10520296909063335

[56] Liu CM, Meinke DW (1998) The *titan* mutants of *Arabidopsis* are disrupted in mitosis and cell cycle control during seed development. The Plant Journal 16: 21–31. 9807824 10.1046/j.1365-313x.1998.00268.x

[57] Backues S.K., Korasick D.A., Heese A., Bednarek S.Y.(2010) The *Arabidopsis* dynamin-related protein2 family is essential for gametophyte development. Plant Cell 22: 3218–3231. 10.1105/tpc.110.077727 20959563 PMC2990125

