# Supplementary figures and images for: Arabidopsis COG Complex Subunits COG3 and COG8 Modulate Golgi Morphology, Vesicle Trafficking Homeostasis and Are Essential for Pollen Tube Growth
Source: PLoS Genet. 2016 Jul 22;12(7):e1006140. doi: 10.1371/journal.pgen.1006140 (PMC4957783; doi:10.1371/journal.pgen.1006140)

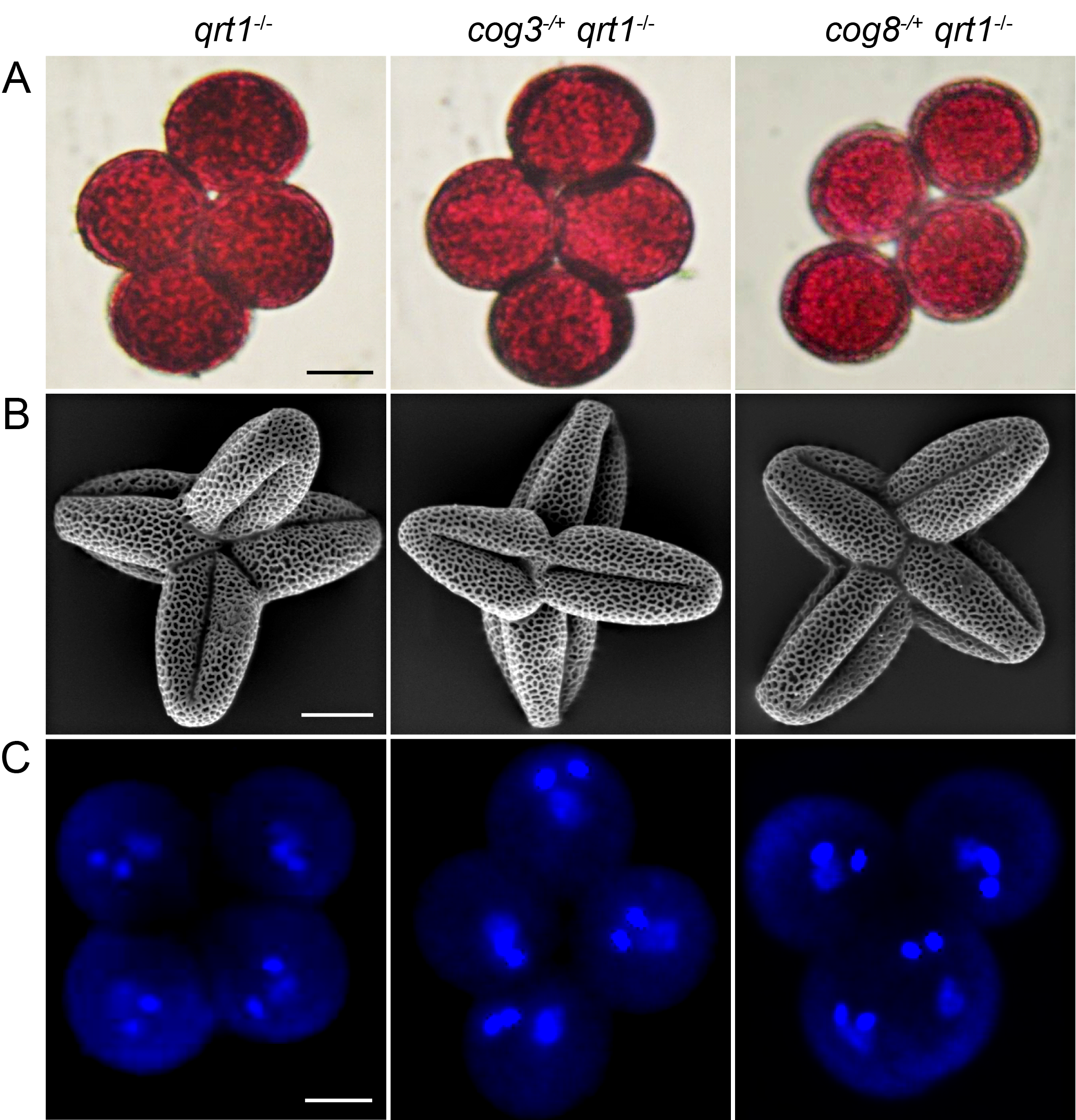

Supplement: S1 Fig — (A) qrt1-/-, cog3-/+ qrt1-/-, and cog8-/+ qrt1-/- quartets exhibit similar Alexander staining patterns. (B) qrt1-/-, cog3-/+ qrt1-/-, and cog8-/+ qrt1-/- quartets are normal in appearance as revealed by scanning electron microscopy (SEM) observation. (C) DAPI staining showing that each mature pollen grain of qrt1-/-, cog3-/+ qrt1-/-, and cog8-/+ qrt1-/- quartets contains two sperm cells and one vegetative cell. Bars = 10 μm in (A), (B), and (C). (TIF) [file pgen.1006140.s001.tif]

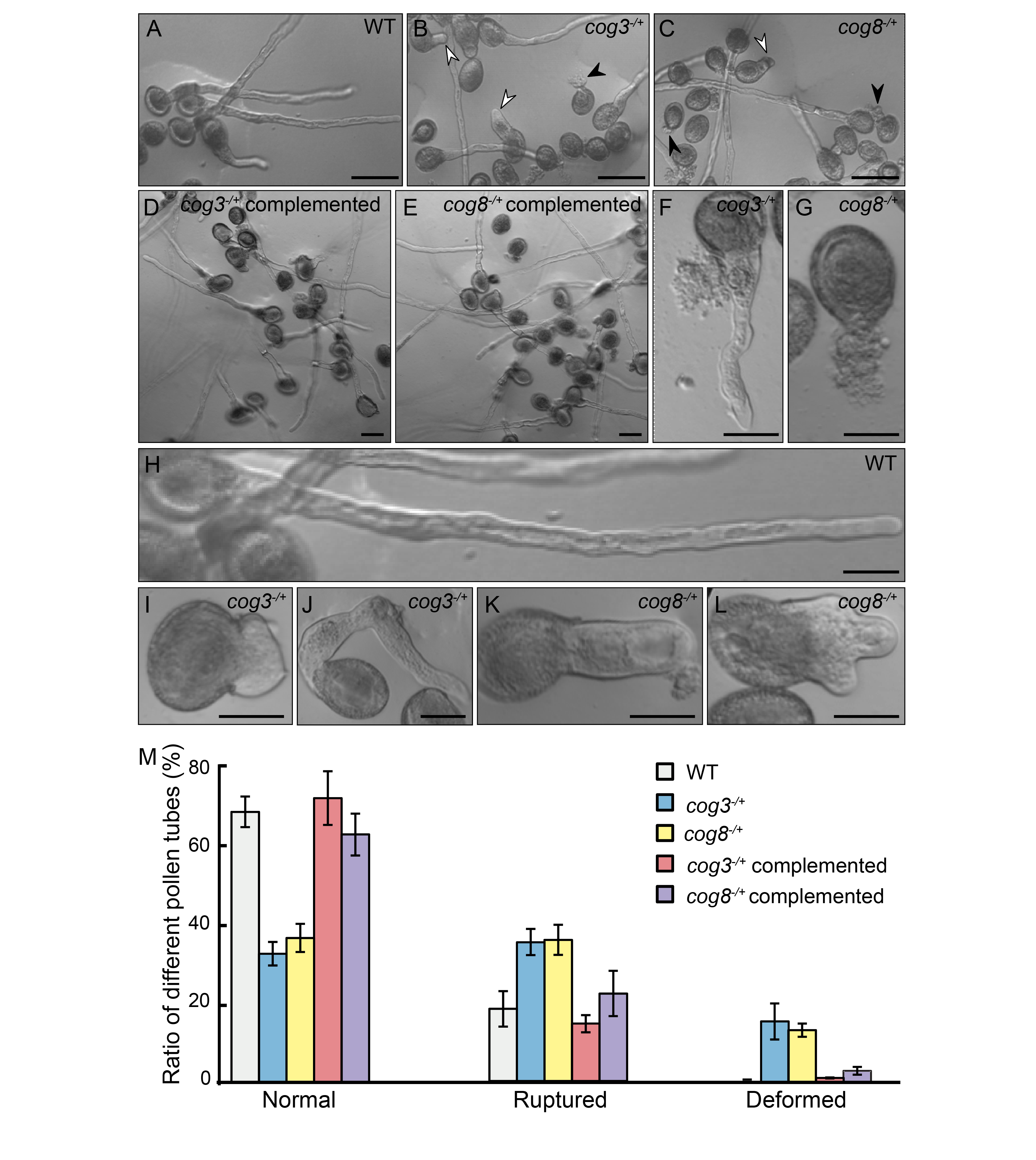

Supplement: S2 Fig — (A), (B), and (C) In vitro growth of wild-type, cog3-/+, and cog8-/+ pollen tubes, respectively. (D) In vitro growth of proLAT52:COG3-GFP complemented cog3-/+ (cog3-/+ proLAT52:COG3-GFP/proLAT52:COG3-GFP) pollen tubes. (E) In vitro growth of proLAT52:COG8-GFP complemented cog8-/+ (cog8-/+ proLAT52:COG3-GFP/proLAT52:COG8-GFP) pollen tubes. (F) and (G) Burst pollen tubes in cog3-/+ and cog8-/+ mutants, respectively. (H) In vitro growth of wild-type pollen tube. (I) to (L) Pleiotropic phenotypes of cog3-/+ and cog8-/+mutant pollen tubes. Short and swollen (I), wavy (J), burst (K), and branched (L) pollen tubes. (M) Statistical analysis of various phenotypes in wild-type, cog3-/+, cog8-/+, proLAT52:COG3-GFP complemented cog3-/+, and proLAT52:COG8-GFP complemented cog8-/+ pollen tubes. n> 500 for each genotype, values represent the means ± SD. Bars = 50 μm in (A) to (C); 20 μm in (D) to (L). (TIF) [file pgen.1006140.s002.tif]

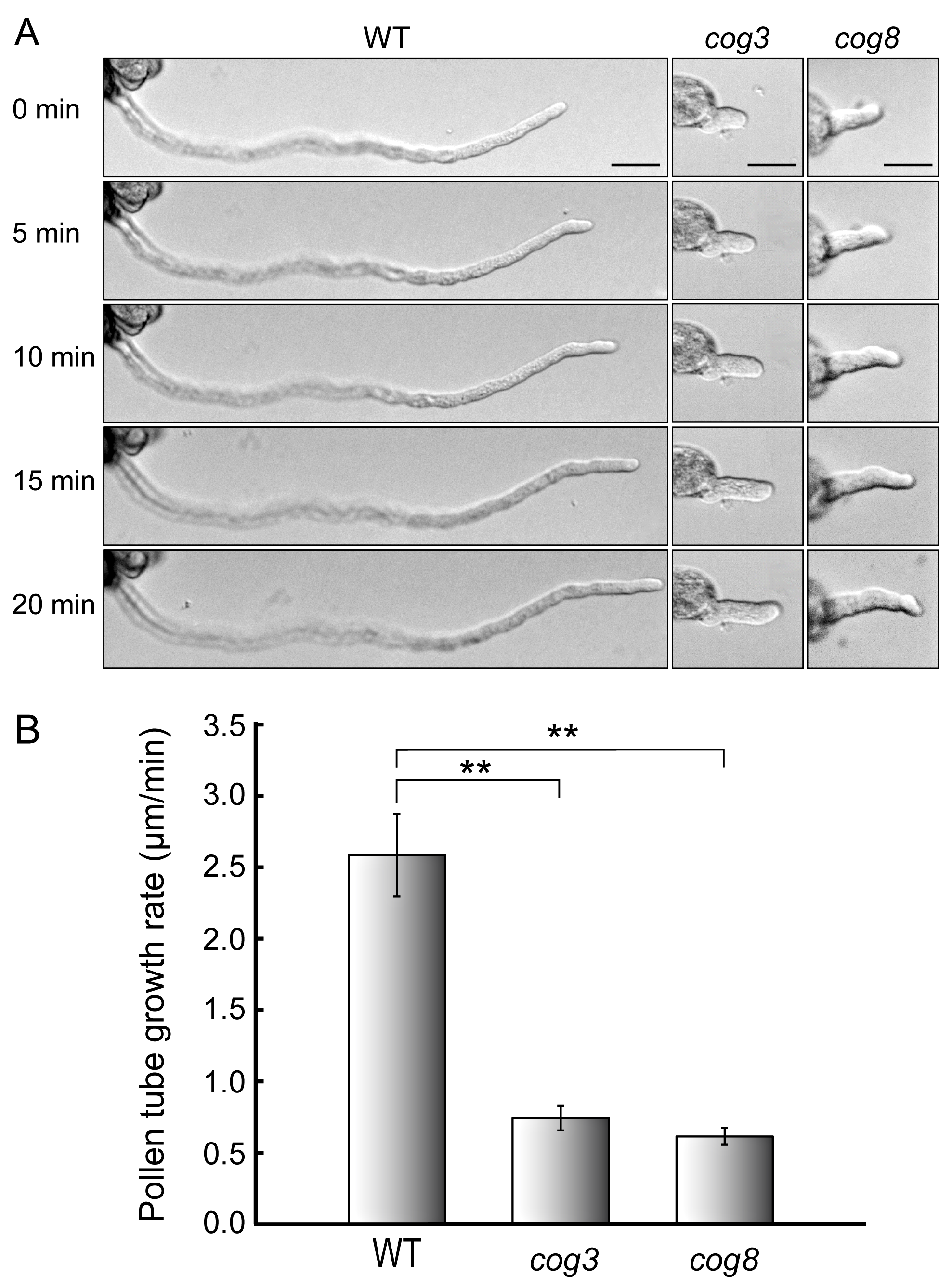

Supplement: S3 Fig — (A) Time-lapse images of wild-type, cog3, and cog8 pollen tubes. (B) Growth of cog3 and cog8 pollen tubes was significantly slower than that of wild-type tubes. Six pollen tubes each of wild-type, cog3, and cog8 were measured. Values represent the means ± SD. **P< 0.001 by Student’s t test. Bars = 20 μm. (TIF) [file pgen.1006140.s003.tif]

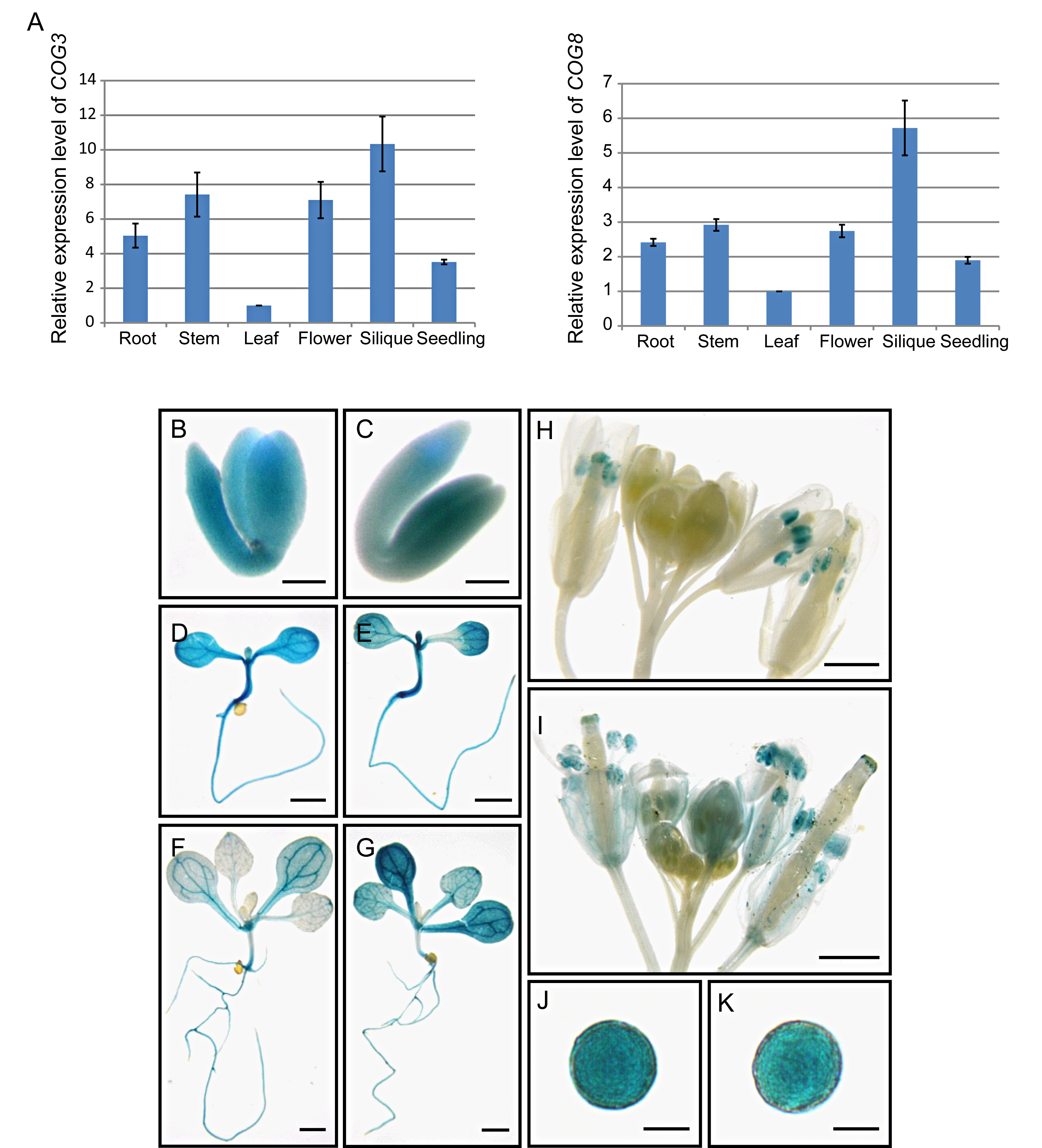

Supplement: S4 Fig — (A) Quantitative RT-PCR assays of COG3 and COG8 genes showed that they are expressed in all tissues examined with the highest expression levels in siliques. (B, D, F, H, J) proCOG3:GUS expression pattern. proCOG3:GUS is expressed in embryos (B), young seedlings (D), seedlings with cauline leaves (F), anthers (H), and mature pollen (J). (C, E, G, I, K) proCOG8:GUS expression pattern in the same tissues examined in proCOG3:GUS plants. Bars = 0.1 mm in (B and C), 1 mm in (D–G), 0.5 mm in (H and I), and 10 μm in (J and K). (TIF) [file pgen.1006140.s004.tif]

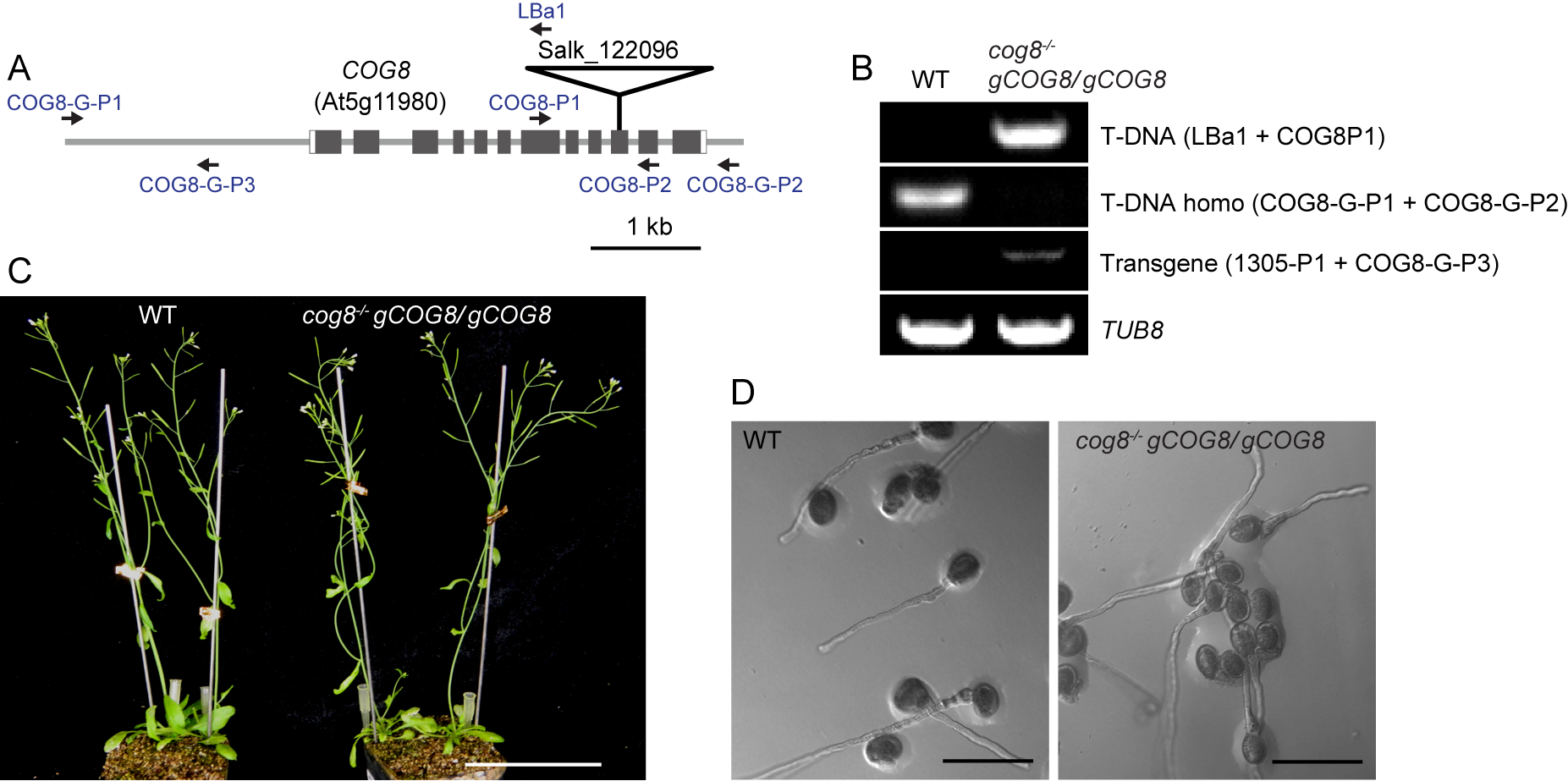

Supplement: S5 Fig — (A) COG8 gene structure and primers used for genotyping. (B) Genotyping of a representative line of COG8 genomic DNA transformed cog8 mutants, which showed hygromycin resistance. Note that the primer1305-P1 located on the vector is not shown here. (C) cog8-/- gCOG8/gCOG8 plants grow normally. (D) Pollen tubes from cog8-/- gCOG8/gCOG8 plants grow normally. Bars = 10 cm in (C), 50 μm in (D). (TIF) [file pgen.1006140.s005.tif]

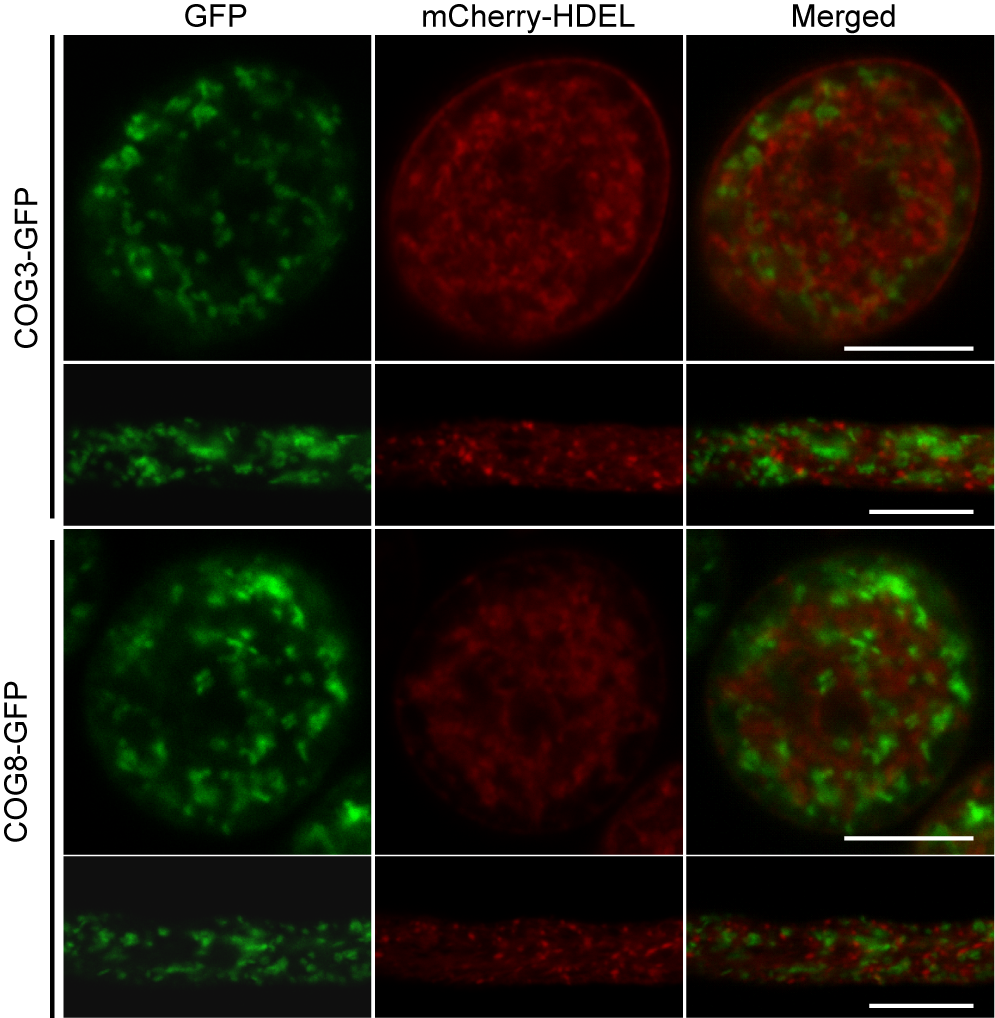

Supplement: S6 Fig — COG3-GFP and COG8-GFP signals are not overlapped with the ER marker mCherry-HDEL in the pollen grains and the pollen tubes. Bars = 10 μm. (TIF) [file pgen.1006140.s006.tif]

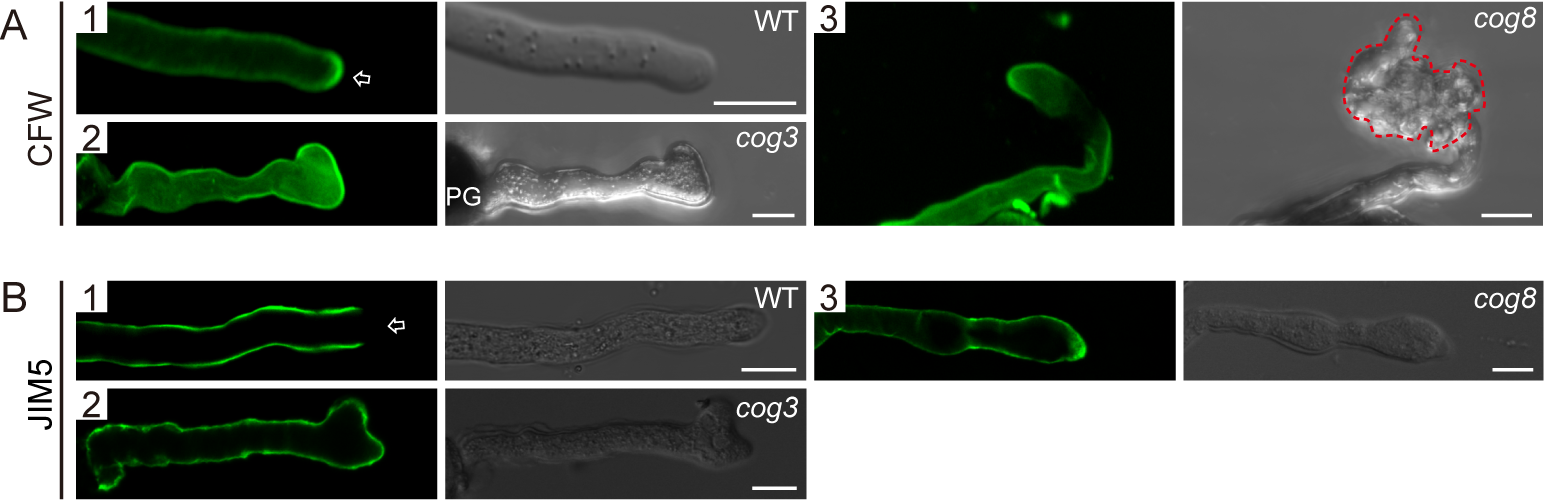

Supplement: S7 Fig — (A) Approximately homogeneous distribution of Calcofluor white staining in the cell wall of a wild-type pollen tube (A1), and similar staining patterns in deformed cog3 and cog8 pollen tubes (A2 and A3). (B) JIM5 epitopes were absent at the tip of a wild-type pollen tube (B1), but present at the tips of cog3 and cog8 mutant pollen tubes (B2 and B3). Yellow lines indicate pollen tube growth directions. Red dashed lines in (A3) highlight the cytoplastic outflow of ruptured cog3and cog8 pollen tubes. Bars = 10 μm. (TIF) [file pgen.1006140.s007.tif]

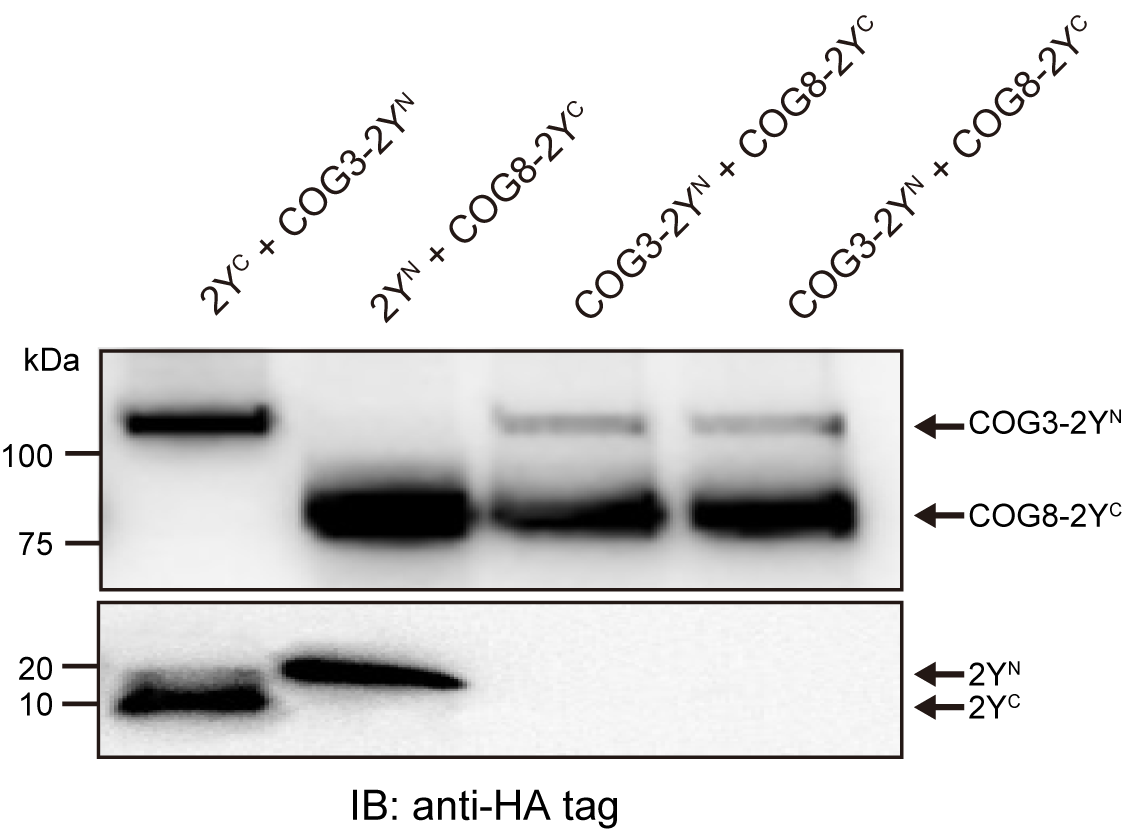

Supplement: S8 Fig — (TIF) [file pgen.1006140.s008.tif]

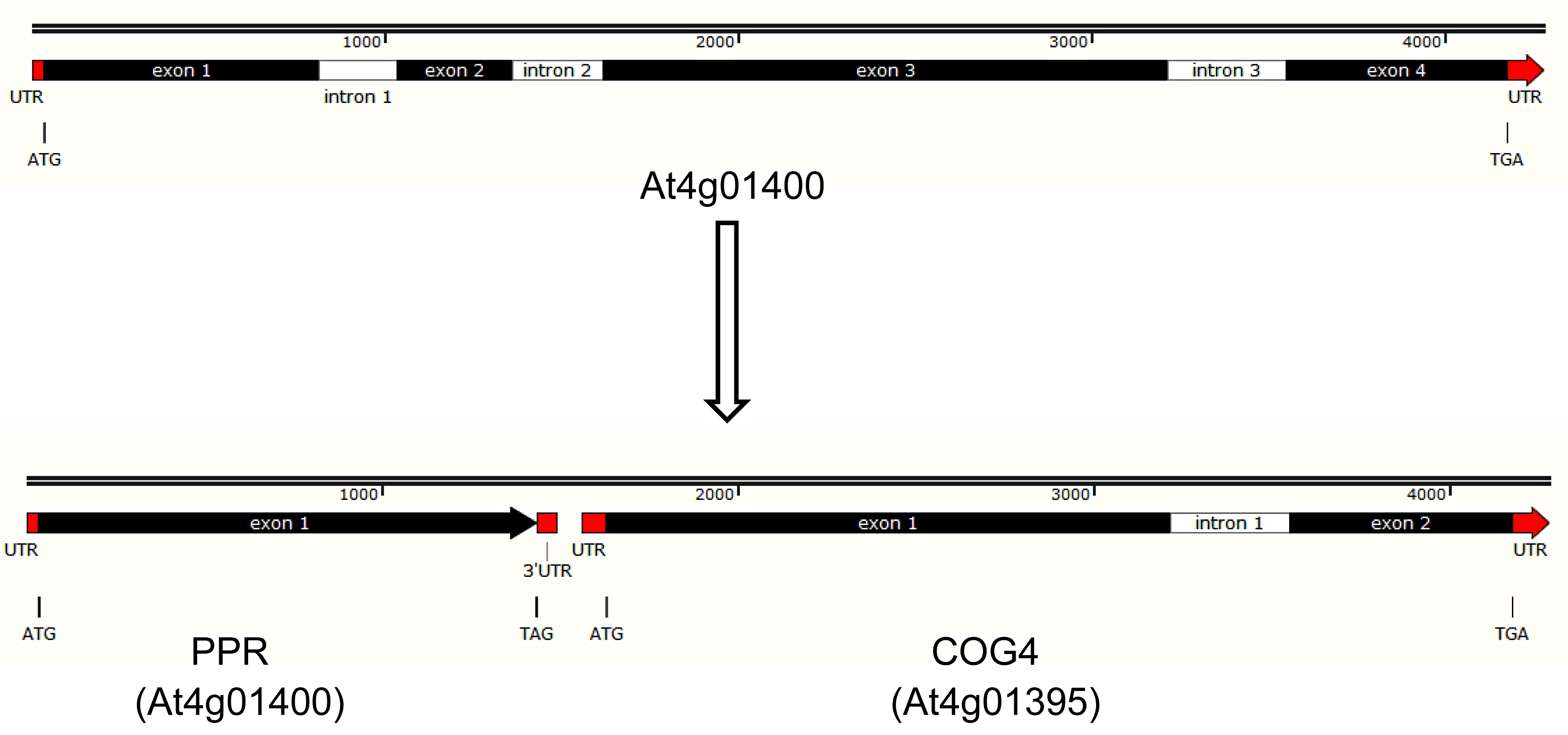

Supplement: S9 Fig — (TIF) [file pgen.1006140.s009.tif]
