# Supplementary material for: Arabidopsis COG Complex Subunits COG3 and COG8 Modulate Golgi Morphology, Vesicle Trafficking Homeostasis and Are Essential for Pollen Tube Growth
Source: PLoS Genet. 2016 Jul 22;12(7):e1006140. doi: 10.1371/journal.pgen.1006140 (PMC4957783; doi:10.1371/journal.pgen.1006140)
Supplement: S1 Table — (DOC) [file pgen.1006140.s010.doc]

**S1 Table.** Complementation analysis of *cog8^-/+^ g^d^COG8^-/+^* lines

| Self-cross | PCR^+a^ | PCR^-b^ | Ratio^c^ | *X^2^*（For 2:1） |
| --- | --- | --- | --- | --- |
| *cog8^-/+^* | 181 | 174 | 1.04 | NA |
| *cog8^-/+^ g^d^COG8^-/+^* (line3) | 127 | 76 | 1.67 | 1.53 |
| *cog8^-/+^ gCOG8^-/+^* (line6) | 138 | 66 | 2.09 | 0.09 |
| *cog8^-/+^ gCOG8^-/+^* (line9) | 136 | 54 | 2.50 | 2.05 |
| *cog8^-/+^ gCOG8^-/+^* (line10) | 145 | 56 | 2.60 | 2.71 |
| ^a^progeny positive for the PCR analysis in the *cog3^-/+^* or *cog8^-/+^* mutant background.  ^b^progeny negative for the PCR analysis in the *cog3^-/+^* or *cog8^-/+^* mutant background.  ^c^Ratio = PCR^+^/PCR^-^  ^d^g is the abbreviation for genomic DNA.  *X^2^* test indicated that the segregation ratio is coincident with expected 2:1 (P<0.05). | | | | |
