# Supplementary material for: Arabidopsis COG Complex Subunits COG3 and COG8 Modulate Golgi Morphology, Vesicle Trafficking Homeostasis and Are Essential for Pollen Tube Growth
Source: PLoS Genet. 2016 Jul 22;12(7):e1006140. doi: 10.1371/journal.pgen.1006140 (PMC4957783; doi:10.1371/journal.pgen.1006140)
Supplement: S2 Table — (DOC) [file pgen.1006140.s011.doc]

**S2 Table.**Characterization of the progeny of *cog3^-/+^* *proLAT52:COG3-GFP/proLAT52:COG3-GFP* and *cog8^-/+^ proLAT52:COG8-GFP/proLAT52:COG8-GFP* mutants.

|  | White seeds | Total seeds  counted | White seeds  (%) | Small seedlings | Total seedlings  counted | Small seedlings  (%) | Total  abnormality  (%) |
| --- | --- | --- | --- | --- | --- | --- | --- |
| *cog3^-/+^*  *proLAT52:COG3GFP/proLAT52:COG3-GFP* | 33 | 192 | 17% | 23 | 210 | 11% | 28% |
| *cog8^-/+^*  *proLAT52:COG8-GFP/proLAT52:COG8-GFP* | 68 | 438 | 16% | 39 | 351 | 11% | 27% |
