# Supplementary material for: Arabidopsis COG Complex Subunits COG3 and COG8 Modulate Golgi Morphology, Vesicle Trafficking Homeostasis and Are Essential for Pollen Tube Growth
Source: PLoS Genet. 2016 Jul 22;12(7):e1006140. doi: 10.1371/journal.pgen.1006140 (PMC4957783; doi:10.1371/journal.pgen.1006140)
Supplement: S3 Table — (DOC) [file pgen.1006140.s012.doc]

**S3 Table.** List of primer pairs used in this study.

| **Usage** | **Primer Name** | **Oligo nucleotide** |
| --- | --- | --- |
| **Genotyping and transgenic plant identification** | COG3-P1 | 5’- GCAACTTTTATGGCTGTG -3’ |
|  | COG3-P2 | 5’- CTTGATTTCTCTCATTTAC -3’ |
|  | GK-LB | 5’- ATATTGACCATCATACTCATTGC -3’ |
|  | COG8-P1 | 5’- CTATCAAATATTTTGGATCAG -3’ |
|  | COG8-P2 | 5’- GTATCTTAGTAAGGAGT -3’ |
|  | COG8-G-P3 | 5’- CAATGATGATGACGATGAGGCC-3’ |
|  | 1305-P1 | 5’- TGGCGAAAGGGGGATGTGCTG-3’ |
|  | COG8-G-P1 | 5’- AAGGAAGAGAGATAGACCAGATCTT-3’ |
|  | COG8-G-P2 | 5’- TCGTACTTTCATCTTTTCATCAG-3’ |
|  | LBa1 | 5’- TGGTTCACGTAGTGGGCCATCG -3’ |
|  | LAT52P1 | 5’- GCATGCCTGCAGGTCGAC -3’ |
|  | LAT52P2 | 5’- GATCCTCTAGACTCCATGG -3’ |
|  | TUB8-F | 5’- CTTCGTATTTGGTCAATCCGGTGC -3’ |
|  | TUB8-R | 5’- GAACATGGCTGAGGCTGTCAAGTA -3’ |
| **Gene expression and protein localization constructs** | pCOG3:GUS-F | 5’- GGGGTCGACGATTAGTTTTTTTGCAGTAACTC -3’ |
|  | pCOG3:GUS-R | 5’- GGGCTGCAGTGTCTATGAAATCTGAATCTC -3’ |
|  | pCOG8:GUS-F | 5’- GGGGTCGACAAGACTCTTCTATATTTTGTATAC -3’ |
|  | pCOG8:GUS-R | 5’- GGGCTGCAGTCCCGGATCTTACTCCTG -3’ |
|  | q-RT COG3-F | 5’- TTATCAAGCATCTCCTTATCT -3’ |
|  | q-RT COG3-R | 5’- CTCTAAGTATCCTCCTCAAG -3’ |
|  | q-RT COG8-F | 5’- TTCCATTGATAAGCATCTC -3’ |
|  | q-RT COG8-R | 5’- CATCTTCCTCTTCTCCAA -3’ |
|  | pLAT52:GFP-F | 5’- CCCGAGCTCATACTCGACTCAGAAGGTAT -3’ |
|  | pLAT52:GFP-R | 5’- GGACTAGTTAATTGGAAATTTTTTTTTTGG -3’ |
|  | pLAT52:COG3-GFP-F | 5’- GGACTAGTATGGCGACTAAGGCGGCT -3’ |
|  | pLAT52:COG3-GFP-R | 5’- GGGCCCGGGAAGGAAATTGTCGAGTTGGG -3’ |
|  | pLAT52:COG8-GFP-F | 5’- GGACTAGTATGGCAATGGAGGTTGG -3’ |
|  | pLAT52:COG8-GFP-R | 5’- GGGCCCGGGAGACTCGGGTGTTATCTC -3’ |
|  | COG8 Genomic-F | 5’- ATTACGAATTCGCAACCGGGTCTGAAGATC -3’ |
|  | COG8 Genomic-R | 5’- AGATCAATTGGATGCAGTCTCAATTC -3’ |
|  | p35S:COG8-GFP-F | 5’- GGACTAGTATGGCAATGGAGGTTGG -3’ |
|  | p35S:COG8-GFP-R | 5’- GGGCCCGGGAGACTCGGGTGTTATCTC -3’ |
|  | pLAT52:γCOP-xFP-F | 5’- CGGATCCATGGCGCAACCCCTCGT -3’ |
|  | pLAT52:γCOP-xFP-R | 5’- GGGTACCGCCGCTGGCAACAATCTC -3’ |
|  | pLAT52:HDEL-mCh-F | 5’- TCTAGAGAGAAGATGAAGGTACAGGAGG -3’ |
|  | pLAT52:HDEL-mCh-R | 5’- GAGCTCCTGCAGTTACAGCTCGTCATG -3’ |
| **Yeast-two-hybridand BIFC vector construction** | pGADT7-COG3-F | 5’- GGAATTCATGGCGACTAAGGCGGCT -3’ |
|  | pGADT7-COG3-R | 5’-TCCCCCGGGGTCGACTTAAAGGAAATTGTCGAGTTGGG -3’ |
|  | pGADT7-COG8-F | 5’- GGAATTCCATATGATGGCAATGGAGGTTGGG -3’ |
|  | pGADT7-COG8-R | 5’- CGGGATCCTCAAGACTCGGGTGTTAT -3’ |
|  | pGBKT7-COG3-F | 5’- GGAATTCATGGCGACTAAGGCGGCT -3’ |
|  | pGBKT7-COG3-R | 5’-TCCCCCGGGGTCGACTTAAAGGAAATTGTCGAGTTGGG -3’ |
|  | pGBKT7-COG8-F | 5’- GGAATTCCATATGATGGCAATGGAGGTTGGG -3’ |
|  | pGBKT7-COG8-R | 5’- CGGGATCCTCAAGACTCGGGTGTTAT -3’ |
|  | COG3-2Y^N^-F | 5’-cccttaattaacATGGCGACTAAGGCGG-3’ |
|  | COG3-2Y^N^-R | 5’-gggactagtAAGGAAATTGTCGAGTTGGGTTTGCA-3’ |
|  | COG8-2Y^C^-F | 5’-cccttaattaacATGGCAATGGAGGTTGGGG-3’ |
|  | COG8-2Y^C^-R | 5’-gggactagtAGACTCGGGTGTTATCTCAGGAGTCTGC -3’ |
|  | pGADT7-COG1-F | 5’- GGAATTCCATATGATGAGAATGTCATCAGCTTC -3’ |
|  | pGADT7-COG1-R | 5’- TCCCCCGGGGTCACTCGGACCTGGTGTT -3’ |
|  | pGADT7-COG2-F | 5’- TCCCCCGGGGATGTCAGATCTGGTCGCGAC-3’ |
|  | pGADT7-COG2-R | 5’-CGGGATCCGTCGACTCAAACACTAATTGAGTTTTGC -3’ |
|  | pGADT7-COG4-F | 5’- GGACTTCCATATGATGATTCGCCGGCCGATC -3’ |
|  | pGADT7-COG4-R | 5’- GGAATTCTCACAACTTGAGAGCAGCAATTGA -3’ |
|  | pGADT7-COG5-F | 5’- GGACTTCCATATGATGGCACTACCTCCATCGTC -3’ |
|  | pGADT7-COG5-R | 5’- GGAATTCTTATAAGTTTTCTTGTGTTAAAGA -3’ |
|  | pGADT7-COG6-F | 5’- GGAATTCATGGCTTCGACGGTGGGA -3’ |
|  | pGADT7-COG6-R | 5’- CGGGATCCTCATATACCAAGAATCGT -3’ |
|  | pGADT7-COG7-F | 5’- GGAATTCATGATGCTGGATCTAGGT -3’ |
|  | pGADT7-COG7-R | 5’- CGGGATCCCTAATCGAAACTGATACG -3’ |
|  | pGBKT7-COG1-F | 5’- GGAATTCCATATGATGAGAATGTCATCAGCTTC -3’ |
|  | pGBKT7-COG1-R | 5’- TCCCCCGGGGTCACTCGGACCTGGTGTT -3’ |
|  | pGBKT7-COG2-F | 5’- TCCCCCGGGG ATGTCAGATCTGGTCGCGAC -3’ |
|  | pGBKT7-COG2-R | 5’-CGGGATCCGTCGACTCAAACACTAATTGAGTTTTGC -3’ |
|  | pGBKT7-COG4-F | 5’- GGACTTCCATATGATGATTCGCCGGCCGATC -3’ |
|  | pGBKT7-COG4-R | 5’- GGAATTCTCACAACTTGAGAGCAGCAATTGA -3’ |
|  | pGBKT7-COG5-F | 5’- GGACTTCCATATGATGGCACTACCTCCATCGTC -3’ |
|  | pGBKT7-COG5-R | 5’- GGAATTCTTATAAGTTTTCTTGTGTTAAAGA -3 |
|  | pGBKT7-COG6-F | 5’- GGAATTCATGGCTTCGACGGTGGGA -3’ |
|  | pGBKT7-COG6-R | 5’- CGGGATCCTCATATACCAAGAATCGT -3’ |
|  | pGBKT7-COG7-F | 5’- GGAATTCATGATGCTGGATCTAGGT -3 |
|  | pGBKT7-COG7-R | 5’- CGGGATCCCTAATCGAAACTGATACG -3’ |
